# Evolution and diversification of Mountain voles (Rodentia: Cricetidae)

**DOI:** 10.1038/s42003-022-04371-z

**Published:** 2022-12-26

**Authors:** Shaoying Liu, Chengran Zhou, Guanliang Meng, Tao Wan, Mingkun Tang, Chentao Yang, Robert W. Murphy, Zhenxin Fan, Yang Liu, Tao Zeng, Yun Zhao, Shanlin Liu

**Affiliations:** 1 Sichuan Academy of Forestry, No.18, Xinhui xilu, Chengdu, 610081 China; 2 BGI-Shenzhen, Shenzhen, 518083 China; 3 Key Laboratory of Bio-Resource and Eco-Environment of Ministry of Education, College of Life Sciences, Sichuan University, Chengdu, 610065 China; 4 Zoological Research Museum Alexander Koenig, D-53113 Bonn, Germany; 5 Reptilia Sanctuary and Education Centre, Concord, ON L4K 2N6 Canada; 6 Centre for Biodiversity and Conservation Biology, Royal Ontario Museum, Toronto, ON M5S 2C6 Canada; 7 Department of Entomology, College of Plant Protection, China Agricultural University, Beijing, 100193 China

**Keywords:** Phylogenetics, Taxonomy, Evolutionary genetics

## Abstract

The systematics of the Cricetid genus *Neodon* have long been fraught with uncertainty due to sampling issues and a lack of comprehensive datasets. To gain better insights into the phylogeny and evolution of *Neodon*, we systematically sampled *Neodon* across the Hengduan and Himalayan Mountains, which cover most of its range in China. Analyses of skulls, teeth, and bacular structures revealed 15 distinct patterns corresponding to 15 species of *Neodon*. In addition to morphological analyses, we generated a high-quality reference genome for the mountain vole and generated whole-genome sequencing data for 47 samples. Phylogenomic analyses supported the recognition of six new species, revealing a long-term underestimation of *Neodon* diversity. We further identified positively selected genes potentially related to high-elevation adaptation. Together, our results illuminate how climate change caused the plateau to become the centre of *Neodon* origin and diversification and how mountain voles have adapted to the hypoxic high-altitude plateau environment.

## Abstract (web summary)

Morphological and molecular phylogenetic analyses reveal six new species of mountain vole from China and climate drivers of mountain vole diversification.

## Introduction

**Fig. 1:**
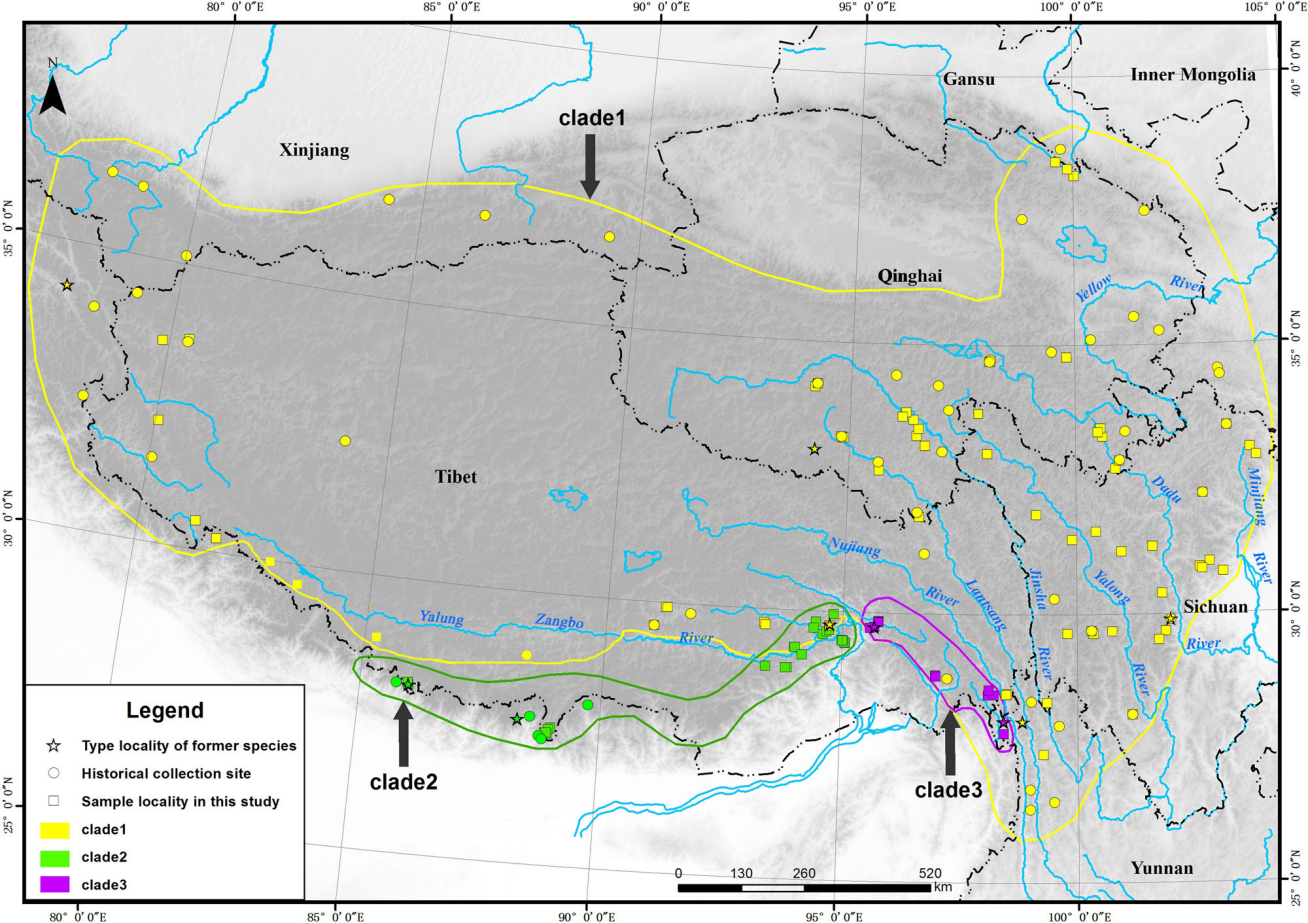
Distribution of *Neodon* used in this study. The approximate extent of occurrence of each clade is shown with coloured lines (Clade 1: yellow; Clade 2: green, Clade 3: purple, refer to Fig. 3 for clade information). Stars show the type localities of the described species. Circles show the historical collection sites. Squares show the distribution of newly collected specimens.

Voles constitute one of the youngest groups of rodents. Mountain voles, belonging to genus *Neodon* (Rodentia: Cricetidae), which occur only in the Tibetan-Himalayan region (THR) (Fig. 1)^1^, originated less than seven million years ago (Mya)^2,3^ owing to the orogenesis of the Hengduan Mountains^4–6^. The THR, comprising of the Himalayas, Hengduan Mountains and the Qinghai-Xizang (Tibet) Plateau (QTP), consists of a series of parallel alpine ridges and deep river valleys and thus shows dramatic ecological stratification and environmental heterogeneity^5,7,8^. The topographical complexity and vastness of the territory constrains fieldwork, which subsequently affects biodiversity estimations. It also affects the testing of hypotheses on species interactions, especially for species with limited dispersal abilities. There is accumulating evidence that the QTP is the centre of origin for many organisms with specific biogeographical relationships to other Palaearctic regions^4,6,9–15^. Thus, systematic sampling with comprehensive analyses in the THR (Fig. 1) can provide critical clues about how geology and climate together drive evolution, biogeography and adaptation.

Previous studies of *Neodon* have been limited by sampling issues. Genus *Neodon* was erected by Horsfield in 1841, with four species (*N. sikimensis, N. irene, N. forresti* and *N. juldaschi*);^16^ however, there has been a long-running taxonomic debate involving the phylogenetic position of *Neodon*, as a subgenus of either *Microtus*^17–19^ or *Pitymys*^20–22^. Recent morphological and molecular evidence confirmed the monophyly of *Neodon*^1,23,24^ and revealed that several *Microtus*-like species once assigned to *Microtus* actually belong to *Neodon*^1,24,25^. Thus, *Neodon* includes far more than four species.

To gain better insight into the diversity, adaptation and evolution of *Neodon*, as well as the roles played by the QTP and climate changes in driving diversification and evolution, we used a collection of more than 2000 specimens collected over the past 20 years from throughout its distribution, especially from the Himalayas. The sampling activities covered tens of thousands of square kilometres, and we analysed the morphology of 235 samples herein (Supplementary Data 1). In addition to morphological and geographic data, we provide a high-quality *Neodon* reference genome obtained using 10X Genomics technology^26^, along with 1X–15X whole-genome sequencing (WGS) data for each morphologically distinct taxon (Supplementary Data 2). This extensive dataset allowed the identification and description of six new species of *Neodon*. Our analyses showed that rapid climate change, complex topography and founder events resulting from dispersal were the key factors driving *Neodon* diversification and evolution. In addition, our de novo genome assembly revealed the genetic basis of the adaptations of mountain voles to high-latitude environments, characterised by pressures such as hypoxia, high UV radiation and low temperatures.

## Results

### Morphological evidence of six unidentified lineages of *Neodon*

We analysed 235 specimens of *Neodon* to examine the possible existence of new species of *Neodon* based on morphological evidence. We also generated WGS data for 48 specimens to explore their genetic divergence and potential taxonomic status (Supplementary Data 1 and 2).

**Fig. 2:**
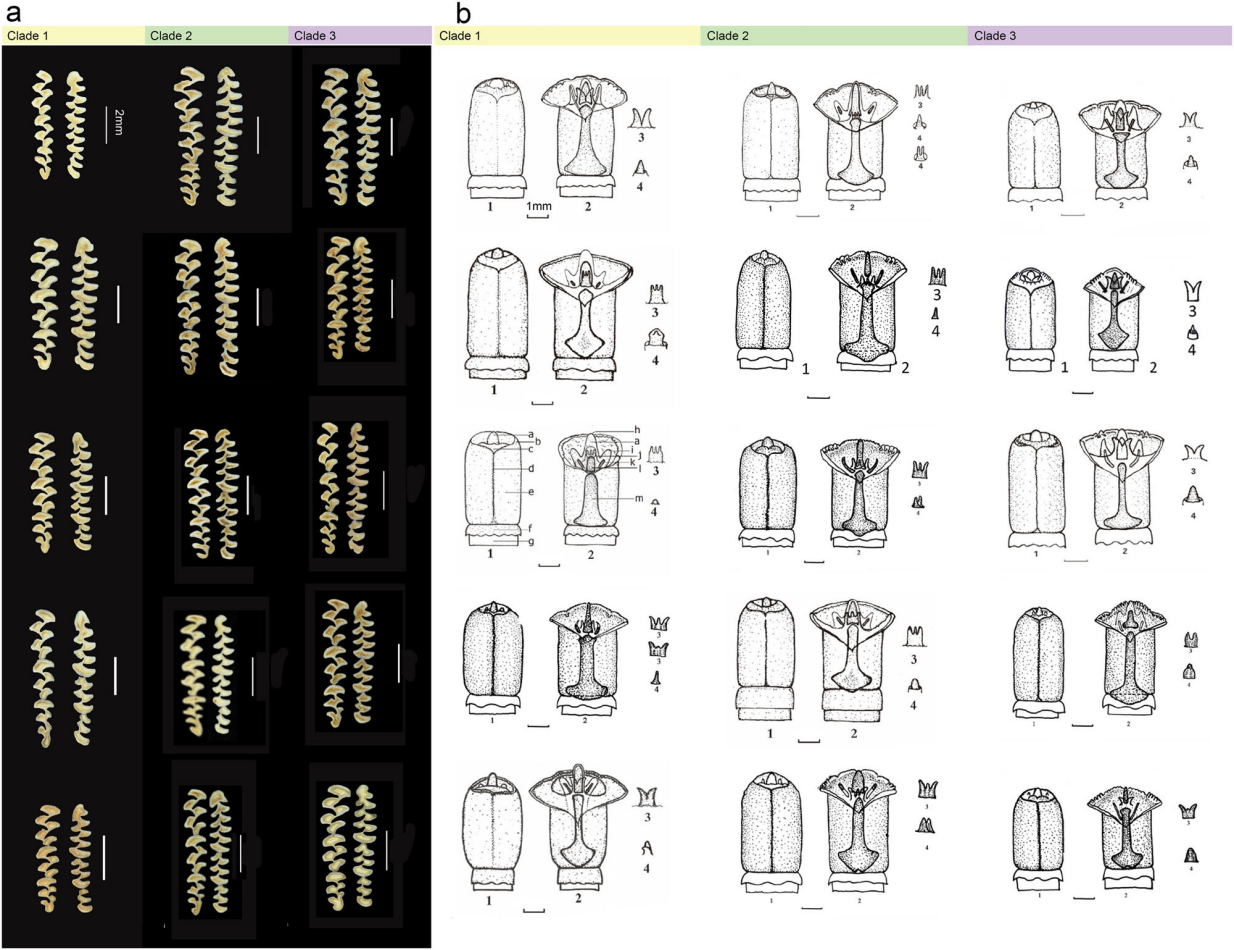
Comparison of morphological features. **a** Comparison of tooth rows. **b** Comparison of glans penes. Numbered views are 1: glans; 2: midventral cut view; 3: urethral lappet; 4: dorsal papilla. Lettered structural features in a1 and a2 are a. distal baculum; b. outer crater; c. inner crater; d. ventral groove; e. glans; f. prepuce; g. penis body; h. station of dorsal papilla; i. lateral baculum (cartilage); j. urethral lappet; k. lateral baculum (bony part); l. distal baculum (bony part); and m. proximal baculum. The taxa are (from top to bottom) *Neodon leucurus*, *N. fuscus*, *N. linzhiensis*, *N. forresti, N. irene* from Clade 1, *N. nyalamensis*, *N. sikimensis*, unidentified taxon 1 (from Nanyi township, Milin County), unidentified taxon 2 (from Shergyla Mountains, Linzhi county), unidentified taxon 3 (from Motuo County, south of the Namchabarwa Mountains) from Clade 2, *N. medogensis*, unidentified taxon 4 (from Ridong village, Bershula Mountains, Chayu County), *N. clarkei*, unidentified taxon 5 (from Bomi County) and unidentified taxon 6 (from Chibagou National Nature Reserve, Chayu County) from Clade 3 (refer to Fig. 3 for clade information).

Initial observations of skulls, teeth and bacular structures showed 15 distinct patterns (Fig. 2 and Supplementary Fig. 1), each representing a putative species of *Neodon*. This included eight described species^1,24^, one previously evaluated group with unclear status and six tentatively unidentified taxa. We recorded the characteristics of the genitalia and bacular structures for males; and dental, external and cranial measurements for both sexes (detailed abbreviations of the measurements are provided in Supplementary Data 3) and further conducted morphological comparisons of these 15 putative species. The morphology of the glans penis provided useful clues about the affinities of microtine species and the differences in the characters of the glans penis clearly distinguished all putative species (Fig. 2). The pairwise Euclidean distances of dental measurements (e.g., the number of closed triangles on the first lower molar) also distinguished 15 patterns (Supplementary Fig. 2 and Supplementary Data 4). In addition, principal component analysis (PCA) (Supplementary Fig. 3) and subsequent two-sided *t*-tests or Wilcoxon rank-sum tests (Supplementary Fig. 4) of 17 statistical measurements of external and cranial characteristics of 95 intact adults (Supplementary Data 5–7) also resolved all 15 putative species.

### Molecular evidence for all lineages of *Neodon*

We generated a total of 241 Gb of 10X Genomics linked-reads (67.56X) for one specimen of *Neodon* sampled from north of the Yarlung Zangbo River on Shergyla Mountain (unidentified taxon 2), and produced a genome assembly with a total length of 2.25 Gb and a scaffold N50 of 10.85 Mb. In addition, we obtained a total of 620.45 Gb of reads for an additional 47 samples, with the amount of data generated from each sample ranging from 2.21 Gb to 43.38 Gb (Supplementary Data 2). A total of 4951 full-length single-copy orthologous gene groups were annotated in our reference genome using BUSCO^27^. The “nuclear gene set” obtained for all lineages after the removal of low-confidence genes, consisted of a total of 4624 coding genes, with an average, maximal and minimal lengths of 1885 nt, 23,046 nt and 222 nt, respectively (Supplementary Fig. 5).

We obtained complete mitogenomes for sequenced specimens and calculated Kimura 2-parameter genetic distances for mitochondrial protein-coding genes (Supplementary Fig. 6 and Supplementary Data 8–10). The results for *cox1* and *cytb*, the two most-widely used barcoding genes in mammals, showed an average interspecific genetic distances of 11.00% for *cox1* and 11.30% for *cytb*. Species delimitation methods (bayesian implementation of Poisson Tree Processes (bPTP), automatic barcode gap discovery (ABGD) and BPP) based on mitochondrial or nuclear datasets recognised the same 15 species, including all six undescribed morphological lineages (Supplementary Figs. 7–10). Furthermore, the species delimitation results identified a split within *N. sikimensis* (Supplementary Fig. 7), and these specimens showed the greatest intraspecific genetic distances (average of 5.23% for *cox1* and 5.20% for *cytb*). However, the specimens of *N. sikimensis*, which were collected in the same region at the same time, did not differ significantly in their morphology. This likely indicated the cooccurrence of divergent mitogenomes, as reported in the Asian elephant^28^ and other species. Further work can explore the possibility of unabated gene flow (one species) or restricted gene flow (two species).

### Phylogenetic analysis corroborates taxonomic status

The analyses generated eight phylogenetic trees, among which two were based on barcoding genes (Supplementary Fig. 11), two were based on 13 mitochondrial coding genes (Supplementary Fig. 7), and four were based on 4,624 nuclear genes (Supplementary Fig. 10). All nuclear trees generated by coalescent and concatenation approaches shared the same topology, with only small-scale incongruences being identified between the nuclear trees and the other four mitochondrial gene trees. We used the nuclear ASTRAL III tree as the species tree in downstream analyses (Supplementary Fig. 10a). The phylogenetic analysis recovered *Neodon* as sister to *Lasiopodomys*, sharing a common ancestor with *Microtus* and *Alexandromys*, and the species of *Neodon* formed three major clades.

### Description of six new species

Multiple resources supported the recognition of six new morphological species. Thus we described these species as follows (Expanded description in Supplementary Note 1, 2). All type series specimens have been deposited with the Sichuan Academy of Forestry:

### *Neodon namchabarwaensis* Liu SY., Zhou CR., Murphy WR. & Liu SL., sp. nov. **(**unidentified taxon 1**)**

#### Holotype

Adult female, field number XZGB0818009, collected by Liao Rui on 10 August 2008. Specimen preserved as a skin, cleaned skull, and tissues. Skull, dentition and mandible in Supplementary Fig. 1a.

#### Type locality

Nanyi township, Milin County, south of Xizang, China, 29.17889° E, 94.15113° N, elevation 3160 m a.s.l.

#### Paratypes

Five specimens topotypes (3♂♂, 2♀♀), field numbers: XZGB0817007♂, XZGB0818006♀, XZGB0818010♂, XZGB0828001♀, XZGB09N235♂;

#### Distribution

Known from south of the Yarlung Zangbo River, north of the Namchabarwa Mountains. The lowest elevation is 3130 m a.s.l.

#### Etymology

Species named for the famous Namcha Barwa Mountain, the highest mountain in this region where the new species occurs.

#### Diagnosis

Medium body, average length 114.9 mm (adult); average hind foot length 20.1 mm. Average tail length 46.4 mm, approximately 40.4% of HBL. First lower molar with 3 closed triangles in front of the posterior transverse space, 6 inner and 5 outer angles. 1st upper molar with 4 inner and 3 outer angles. 2nd upper molar with 3 inner and 3 outer angles. 3rd upper molar with 4 inner and 3 outer angles.

### *Neodon shergylaensis* Liu SY., Zhou CR., Murphy WR. & Liu SL., sp. nov. **(**unidentified taxon 2**)**

#### Holotype

Adult male, field number XZGB09N195, collected by Liao Rui and Liu Yang on 30 May 2009. Specimen preserved as a skin, cleaned skull, penis and tissues. Skull, dentition, and mandible are in Supplementary Fig. 1b.

#### Type locality

Shergyla Mountains of Linzhi county, southeast of Xizang, China, 29.62368° E, 94.66174° N, elevation 4500 m.

#### Paratypes

6 specimens (1♂, 5♀♀), field numbers: LZRAP01013♂, LZRAP01020♀, LZRAP01014♀, LZRAP01019♀, XZGB09N197♀, GB0815001J♀

#### Distribution

Known from north of the Yarlung Zangbo River at over 3160 m a.s.l. both sides of Shergyla Mountains and Niyang River.

#### Etymology

The species is named for its type locality. This region supports a high biodiversity.

#### Diagnosis

Medium body, average length 115.7 mm (adult); average hind foot length 19.5 mm. Average tail length 42.7 mm, approximately 37% of HBL. The first lower molar with 3 closed triangles in front of the posterior transverse space, 6 inner and 4 outer angles in 63% specimens, 6 inner and 5 outer angles in 37% specimens. 1st upper molar with 3 inner and 3 outer angles. 2nd upper molar with 3 inner and 3 outer angles. 3rd upper molar with 4 inner and 3 outer angles.

### *Neodon liaoruii* Liu SY., Zhou CR., Meng GL. & Liu SL., sp. nov. **(**unidentified taxon 3**)**

#### Holotype

Adult male, field number XZ11117, collected by Liao Rui on 1 November 2011. Specimen preserved as a skin, cleaned skull, penis and tissues. Skull, dentition and mandible in Supplementary Fig. 1c.

#### Type locality

Motuo County, south of Xizang, China, 29.47028° E, 94.984° N, elevation 3260 m.

#### Paratypes

Ten specimens (4♂♂, 6♀♀), field numbers: MT11036♀, MT11066♀, MT11067♀, MT11109♂, MT11118♂, MT11120♂, MT11122♀, MT11142♂, MT11143♀, MT11144♀.

#### Distribution

Known from south of the Namchabarwa Mountains. Lowest elevation 2660 m a.s.l.

#### Etymology

Species epithet is a patronym for the collector, Mr. Liao Rui. He made an important contribution to our collecting specimens.

#### Diagnosis

Relatively large body, average length 116.8 mm (adult); average hind foot length 21.1 mm. Avarage tail length 59.3 mm, ~50.8% of HBL. First lower molar with 3 closed triangles in front of the posterior transverse space, 6 inner and 5 outer angles. 1st upper molar with 3 inner and 3 outer angles. 2nd upper molar with 2 inner and 3 outer angles in 66% specimens, and 3 inner and 3 outer angles in another 34% specimens. 3rd upper molar with 4 inner and 3 outer angles in 61% specimens, and 3 inner and 3 outer angles in another 39% specimens.

### *Neodon bershulaensis* Liu SY., Zhou CR., Liu Y. & Liu SL., sp. nov. **(**unidentified taxon 4**)**

#### Holotype

Adult male, field number XZ11010, collected by Liao Rui on 3 Mach 2011. Specimen preserved as a skin, cleaned skull, penis and tissues. Skull, dentition, and mandible in Supplementary Fig. 1d.

#### Type locality

Ridong village, Bershula Mountains, Chayu County, southeast of Xizang, China. 98.12407° E, 28.58392° N, elevation 3750 m a.s.l.

#### Paratypes

3 intact adult specimens, field number: CHYRD-03♀, CHYRD-04♂, CSD3825♂.

#### Distribution

Known from the type locality only, Ridong village, Chayu County, southeast of Xizang.

#### Etymology

Species epithet for the famous Bershula Mountains, where type locality, Ridong is at its foot.

#### Diagnosis

Medium body, average length 107 mm (adult); hind feet length 18–20 mm (average 19 mm). Average tail length 51.5 mm, 48.1% of HBL. First lower molar with 5 closed triangles in front of the posterior transverse space, 6 inner and 4 outer angles. 1st upper molar with 4 inner and 3 outer angles in 70% specimens; other 30% with 3 inner and 3 outer angles. 2nd upper molar with 3 inner and 3 outer angles. 3rd upper molar with 4 inner and 3 outer angles.

### *Neodon bomiensis* Liu SY., Zhou CR., Meng GL. & Liu SL., sp. nov. **(**unidentified taxon 5**)**

#### Holotype

Adult male, field number XZ13015, collected by Liao Rui on 31 October 2013. Specimen preserved as a skin, cleaned skull, penis and tissues. Skull, dentition and mandible in Supplementary Fig. 1e.

#### Type locality

Bomi County, southeast of Xizang, China, 95.9575816° E, 29.82959° N, elevation 2900 m a.s.l.

#### Paratypes

2 intact adults specimens, field numbers: MT11304♀, XZ13016♀

#### Distribution

Known only from the type locality, Bomi County, southeast of Xizang.

#### Etymology

Species epithet derived from the county where type series collected.

#### Diagnosis

Medium body, average length 111.75 mm (adult); hind feet length 18–19 mm (average 18.75 mm). Tail length 53–56 mm (average 53.75 mm), approximately 48.1% of HBL. First lower molar with 4 closed triangles in front of the posterior transverse space, 6 inner and 4 outer angles in 60% specimens; other 40% with 5 inner and 4 outer angles. 1st upper molar with 4 closed triangles after the anterior transverse space, forming 3 inner and 3 outer angles. 2nd upper molar with 3 inner and 3 outer angles, and the last inner angle much small. 3rd upper molar with 3 inner and 3 outer angles.

### *Neodon chayuensis* Liu SY., Zhou CR., Liu Y., Tang MK. & Liu SL., sp. nov. **(**unidentified taxon 6**)**

#### Holotype

Adult female, field number CY37, collected by Liu Yang on 8 October 2007. Specimen preserved as a skin, cleaned skull and tissues. Skull, dentition, and mandible in Supplementary Fig. 1f.

#### Type locality

Chibagou National Nature Reserve, Chayu County, southeast of Xizang, China, 96. 98858° E, 28. 85716° N, elevation 2960 m a.s.l.

#### Paratypes

Three intact adults specimens (1♂, 2♀♀), field numbers: CY35♀, CY44♂, CY45♀.

#### Distribution

Known from Cibagou National Nature Reserve, Chayu County, southeast of Xizang.

#### Etymology

Species epithet derived from the county where type series were collected.

#### Diagnosis

Medium body, average length 106.6 mm (adult); hind feet length 19–21 mm. Average tail length 47.9 mm, approximately 45% of HBL. Tooth row sturdy. First lower molar with 4 closed triangles in front of the posterior transverse space, but more or less confluent each other in many specimens, this tooth with 6 inner and 5 outer angles in 55% specimens; other 45% specimens with 6 inner and 4 outer angles. 1^st^ upper molar with 4 inner and 3 outer angles in 67% specimens, another 33% with 3 inner and 3 outer angles. 2^nd^ upper molar with 3 inner and 3 outer angles. 3^rd^ upper molar with 3 inner and 3 outer angles.

### Historical biogeography of mountain voles

**Fig. 3:**
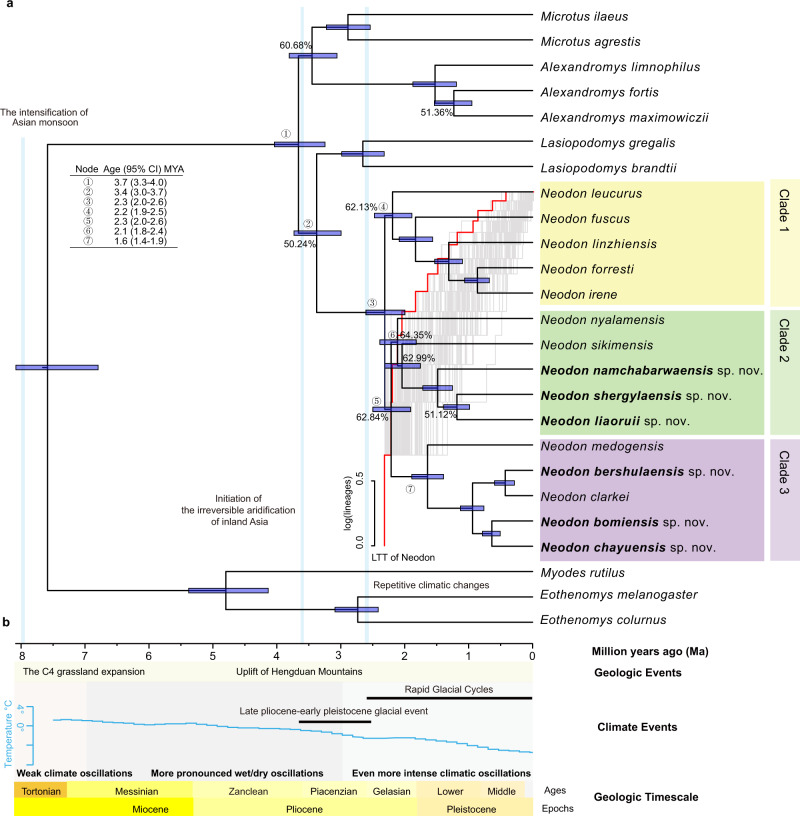
Divergence time tree, diversification patterns, the frequency of the non-main topologies and photos of *Neodon*. **a** Divergence time-tree with the Astral branch supports values of the nuclear gene tree are shown near the branches. New species denoted in bold. Clades of *Neodon* indicated by shading with different colours on the tree (Clade 1: yellow; Clade 2: green; Clade 3: purple). The blue rectangles at the nodes represent 95% confidence intervals of the corresponding estimated divergence times. Branches with high non-main topology occurrence frequencies (>50%) are marked with the content number. Log-lineage-through-time (LTT) plots for *Neodon* estimated from the time-calibrated phylogeny (red curve), and the semilucent red dashed line indicates the null distribution under a Yule process. **b** The divergence time, geologic timescale, geologic events and climate events are shown at the bottom of the figure.

**Fig. 4:**
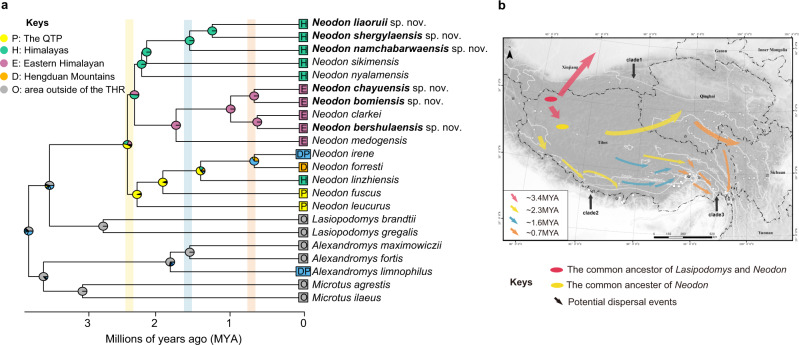
Ancestral range estimation of *Neodon*. **a** Ancestral range estimation based on the best-fitting model DEC + J implemented in BioGeoBEARS. The relative probabilities of each ancestral reconstruction are indicated with pie charts at each node. New species denoted in bold. Transparent bars represent the timelines following the coding described in b. **b** Estimated origin and dispersal of extant *Neodon*.

The divergence of *Neodon* and *Lasiopodomys* at ca. 3.4 Mya (3.7–3.0 Mya) coincided with the onset of the Late Pliocene-early Pleistocene glacial event, and most lineages of *Neodon* have diverged over the past 3 million years (Fig. 3). The newly identified species belonged mainly to Clades 2 and 3. The short branch lengths of the inner nodes revealed that *Neodon* experienced an explosive radiation during the late Neogene and that its speciation occurred very quickly (Fig. 3). Both the lineage-through-time plot and gamma statistic (*γ* < 0, *p* < 0.05) suggested a deceleration of the rate of speciation after the initial burst (Fig. 3 and Supplementary Fig. 12). The best-fitting model was dispersal-extinction cladogenesis with a long-distance J parameter (DEC + J) based on BioGeoBEARS analysis (Supplementary Data 11). The most recent common ancestor of *Neodon* was dated to approximately 2.6 Mya and it was located on the QTP with the highest probability (Fig. 3). Within the next two million years, glacial cycles and climate oscillations increased and caused more uncertainty in the environment (Fig. 3b). The ancestor of each clade appeared to have dispersed to plateaus, such as those in the Himalayas and Hengduan Mountains. Speciation involved different niches within the plateau and surrounding mountains, suggesting that the QTP is the centre of origin of mountain voles (Fig. 4). Our phylogeny demonstrates that the evolution of mountain voles has involved several critical geographical and climate events.

### Positively selected genes

**Fig. 5:**
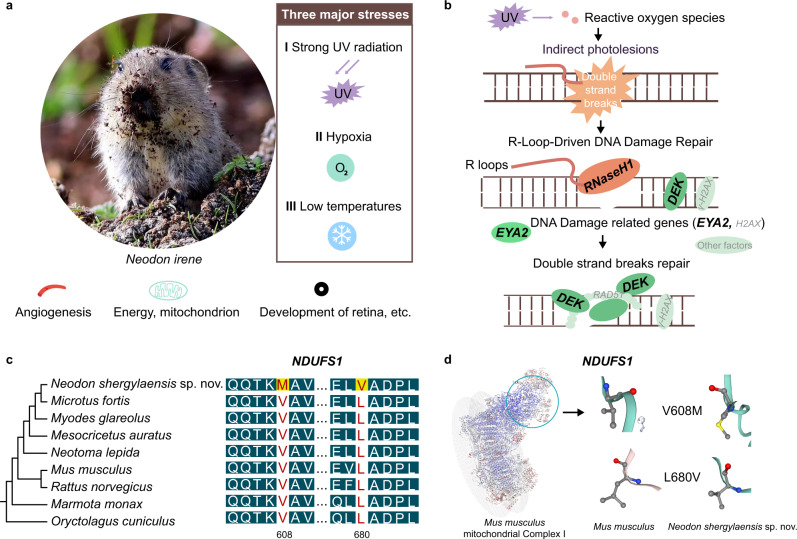
Gene functional adaptations in *Neodon* species. **a** The three major environmental stresses of high-elevation areas and the adaptive traits that the voles may have developed (Photograph by Shaoying Liu). **b** The repair of DNA double-strand breaks damage, involving the three PSGs of RNaseH1, EYA2 and *DEK*, is a classic DNA repair pathway for UV-induced DNA damage. **c** Visualisation of two positively selected gene sites in the *NDUFS1* gene, which represents one of the PSGs related to mitochondrial function. **d** Mitochondrial complex I and positive selection sites affect the protein structure of *NDUFS1*.

High-elevation mammals show many adaptations to hypoxia, low temperatures and high levels of ultraviolet radiation^15,29^. Hence, we investigated the plateau adaptation of mountain voles based on 6,678 high-confidence orthologous genes of *Neodon shergylaensis s*p. nov. (our *de novo* genome) and eight other low-elevation Glires taxa (Fig. 5). We identified 127 positively selected genes (PSGs) (Supplementary Data 12) using the PAML branch-site model^30^. These genes were associated with physiological processes that may contribute to adaptations such as DNA repair (*RNASEH1*, *EYA2*, *DEK*)^31–33^, eye development (R*AB25, WSCD2, MYO7A, MEGF11, RPE65*), skin and fur (*KRTAP5-1, KRTAP4-6*), energy production or mitochondrion development (*NDUFS, MPZL3*, *TRAK1*, *REEP1)*^34,35^, angiogenesis (*MST1*, *PHACTR1, CHRM2*)^36^, and the somatosensory system^37^ (*IGF2BP2*, *RHBDF1*). Furthermore, KEGG and GO enrichment analyses of the PSGs revealed “Retinol metabolism” as a significant process (Supplementary Data 13, 14), which suggested that mountain voles have evolved specific mechanisms to protect their eyes and skins from the risks of UV-induced tumours or other diseases^38^. Genes that function in energy production, angiogenesis and the somatosensory system (Fig. 5 and Supplementary Data 13–15) may have contributed to adaptations by increasing oxygen delivery via adaptive vasodilation^39–41^ and low-temperature resistance^42^.

## Discussion

The morphological traits of the penes and dentition, and external and cranial characters are critical for classifying species in tribe Arvicolini^1,13^ and vary among species of *Neodon*. For example, features of the glans penis and baculum offer the most valuable clues for identification (Fig. 2), while the number of closed triangles on the first lower molar, which has been used to characterise diversity in *Neodon*^13,43,44^, does not explain the full spectrum of morphological diversity. All specimens with three closed triangles in the first lower molar (distributed in southern Tibet) were once regarded as *N. sikimensis*^1,24^, but our results reveal that at least five species share the same feature, including *N. sikimensis, N. nyalamensis, N. liaoruii* sp. nov.*, N. shergylaensis* sp. nov. and *N. namchabarwaensis* sp. nov. It is possible to identify the species of *Neodon* by combining external measurements and other morphological traits of the upper and lower molars, such as the number of inner and outer angles.

Climate oscillations and geographical events appear to have played crucial roles in *Neodon* speciation. For instance, global cooling and the irreversible aridification of inland Asia coincided with the divergence of *Neodon* and *Lasiopodomys*: the divergence of the common ancestor species of the two groups occurred in the late Pliocene (~3.4 Mya), when the Earth’s surface was generally cooled (Fig. 3b). Orogenesis in the THR imposed a large barrier effect on the ancient southwestern monsoons, which directly resulted in the South Branch Westerly Jetstream and further promoted the transformation of the frigid and rough environment on the plateau surface into a humid environment^45,46^. The common ancestor of *Lasiopodomys* and *Neodon* appears to have differentiated as it adapted to different climates. One lineage adapted to drought conditions and grassland environments appears to have retreated to the arid north plateau and evolved into the *Lasiopodomys*, while another branch, adapted to humid environments, occupied the humid plateau surface and evolved into *Neodon* (Fig. 4b, magenta arrows). Then, as the last Ice Age began at ~2.6 Mya^47^, the climate cooled and forced the ancestral population of *Neodon* to migrate to the southern or eastern edge of the THR and then return to the inner THR after the ice sheet receded, which resulted in the speciation of many Tibetan species^48^.

A further inspection of the geographic distributions and barriers of those species showed that the lower Himalayan foothills, Hengduan Mountains and Yalung Zangbo River played vital roles in *Neodon* speciation. The two most widely distributed species of *Neodon*, *N. leucurus* and *N. fuscus*, were located at the basal position of the *Neodon* species tree (Clade 1, Fig. 3 and Supplementary Figs. 13 and 14), confirming that *Neodon* originated on the QTP. The species in Clade 2 included two described and three newly discovered taxa distributed mainly in the Himalayas (Fig. 3 and Supplementary Fig. 15). *Neodon sikimensis* and *N. nyalamensis* specimens collected near their type localities formed a root lineage with other members of Clade 2. The three new taxa occured around the Yalung Zangbo River, Namchabarwa Mountains and Duoxiongla Peak, which were not previously explored. The species of Clade 3 live in the eastern THR (including the Hengduan Mountains and the eastern margins of the QTP), where mountain ranges or single summits could have served as glacial isolated island refugia (Fig. 3, Supplementary Fig. 16)^4,49^. Furthermore, the Yarlung Zangbo River and Palung Zangbo Ancient River Channel appear to have formed physical barriers that facilitated the genetic divergence between *N. nyalamensis, N. medogensis* and other species (Supplementary Figs. 14–16).

The distributions and phylogenetic positions of the species suggest that Pleistocene glacial periods drove dispersal from the inner QTP to refugia in the Himalayas, Hengduan Mountains and montane regions isolated by intervening valleys^4,49^. The complex topographical features led to the geographic isolation of biota. An overall positive correlation existed between genetic distances and geographic distances, but several geographically close populations did not follow this pattern (Supplementary Figs. 14–16). The pattern likely matches with sky island refugia distribution located along the dispersal route. Furthermore, the dispersal route of *Neodon* closely corresponded to with those of other species, such as the woolly rhinoceros^50^, dipodids^11^ and pikas^15^. A strong negative correlation (*R*^2^ = 0.93, *P* value < 0.01) was identified between the frequency of the non-main topologies (the two alternative topologies) and inner-node branch length (Supplementary Fig. 12), indicating that potentially high levels of incomplete linage sorting or gene flow played a role in the initial rapid species radiation (Supplementary Fig. 12c). This result implies that genetic drift drives most molecular changes in lineages of *Neodon* and that the species still share similar ecological niches but present different morphologies.

The genome resources will provide better insight into the adaptation and evolution of *Neodon* and additional THR species. The analyses revealed 127 orthologous genes that have undergone positive selection. Gene function analyses revealed involvement in multiple physiological processes that facilitate survival on the QTP. These genetic signatures of adaptation provide important insights for understanding of how the genomes have changed during high-elevation adaptation. Nevertheless, the confirmation of adaptation awaits functional testing.

Morphological, genetic and biogeographic evidence allows the exploration of the history of *Neodon*’s evolution. Our results offer a more complete understanding of speciation and biogeographic events and further highlight how mountain voles adapted to new environments of the THR during climate events. Most of the newly described species came from localities that were not sampled previously. Thus, similar sampling and integrative molecular and morphological analyses of relatively sedentary species, such as small mammals, reptiles and amphibians, are necessary to accurately document the biodiversity of the THR. Similar investigations of other small mammals will likely reveal greater diversity and ultimately identify the common driver(s) of patterns in the THR ecosystem, and further revealing the evolutionary history of these animals and elucidating the genetic basis of their adaptation toward extreme ecological preferences.

## Methods

### Sample information

We analysed up to 235 specimens of *Neodon* (Supplementary Data 1). All samples were obtained following the Guidelines of the American Society of Mammalogists^51^ and the laws and regulations of China for the implementation of the protection of terrestrial wild animals (State Council Decree 1992). Voucher specimens were deposited in the Sichuan Academy of Forestry, Chengdu, China.

### Morphological analyses

External measurements were recorded in the field from all freshly captured specimens at an accuracy of 0.5 mm, and the cranial and dental characteristics of all specimens were measured using a Vernier calliper with an accuracy of 0.02 mm in the lab (Supplementary Data 3)^1^. The morphometric variation in 17 non-sex-related measurements of adult specimens was analysed using PCA in SPSS v17.0 (SPSS Inc., Chicago, IL, USA). We employed Kaiser-Meyer-Olkin^52^ and Bartlett’s tests^53^ to check the fitness of the PCA, followed by *Tukey’s test*^54^. Independent-samples two-sided *t*-tests or Wilcoxon rank-sum tests^55^ were also performed to check the differences between the taxon pairs after PCA. Plots were generated using R corrplot^56^, ggplot2^57^ and Python3.

### High-throughput sequencing and genome assembly

We extracted the genomic DNA of each specimen from muscle tissues using a Gentra Puregene Tissue Kit (Qiagen, Valencia, CA) according to the manufacturer’s protocol and then generated >10 Gb of data for most morphologically distinct species and ~3 Gb of data (~1X) for the other specimens of the same species. In addition, we sequenced *Neodon shergylaensis* sp. nov. using 10X technology to obtain ~241 Gb of data (~86X). Low-quality reads were removed if they met one or more of the following criteria: (1) an N-content of more than 10%; (2) the presence of adaptor contamination (reads overlapping more than 50% with the adaptor sequence, with a maximal 1 bp mismatches to the adaptor sequence); or (3) more than 30% of the read length below Q30 (Supplementary Data 2).

A total of 1,612.36 million paired-dnd reads were generated with 10X technology was generated for *Neodon shergylaensis* sp. nov., and the reference genome was assembled using SuperNova v2.1.1^58^, with a preset genome size of 2.50 Gb and a weighted mean molecule size of 18.39 kb. One of the two pseudohaplotypes generated using SuperNova with “*--maxreads* = *‘all’ --accept-extreme-coverage, --style* = *pseudohap2*” was used to obtain the core gene set using BUSCO v3.0.1^27^. In addition, the assembled genome was annotated using MAKER2 v2.31.10^59,60^ (control files can be found in Supplementary Data 16) for further evolutionary analysis. We also assembled and annotated the mitogenome of each sample using MitoZ^61^ with ~3 Gb (~1X) of filtered data^61^.

### Gene dataset construction

A read-mapping-then-consensus-calling pipeline was used to obtain orthologous nuclear genes for each sample. For this purpose, (1) we obtained complete and single-copy orthologues of the *N. shergylaensis* sp. nov. genome using BUSCO v3.0.1^27^ with a database of the Euarchontoglires group (6192 genes, v2), and we removed orthologues with high homologue to each other (BLASTn v2.6.0+ with *e* value < 1e-5)^62^ to avoid mapping uncertainty in the subsequent steps, deleted orthologues with internal stop codons, extracted corresponding genomic regions for the remaining qualified orthologues with custom Python scripts and then used the residual data as a reference for the resequenced samples. (2) We mapped the WGS data of each sample onto the reference using BWA-MEM v0.7.17^63^ with the default parameters, the corresponding genes were acquired using the consensus calling function in BCFtools v1.8^64^ (detailed in Supplementary Note 1) and then selected high-quality coding sequences (no internal stop codons, ‘N’ content below 20%, present in more than 50% of samples) for subsequent analyses. (3) To obtain gene alignments, we applied MAFFT v7.313^65^ to generate the multiple protein sequence alignment for each qualified orthologue and then obtained the corresponding gene alignments based on the protein alignments using PAL2NAL^66^ for mitochondrial genes and TrimAl v1.4.rev22^67^ with the parameters “-backtrans -automated1” for nuclear genes. All multiple sequence alignments are provided in the Supplementary Data 17.

To evaluate the potential bias of the reference-mapping-based (“mapping-derived”) method, we obtained the orthologues of six other high-coverage sequencing samples using a *de novo* assembly method. We compared the exon datasets obtained from the “mapping-derived” method and the “de novo-derived” methods, and calculated the mismatches between them. We additionally subsampled the clean read dataset and added three more filtering parameters for the mapping-derived method for comparison. The details are provided in Supplementary Note 1 (Supplementary Fig. 17 and Supplementary Data 18 and 19). The “mapping-derived” gene dataset was used in subsequent analyses.

### Species delimitation

We calculated the Kimura 2-parameter genetic distances between lineages for each gene using the dist.dna function in R ape v1.1-1^68^ and explored the correlation between genetic distance and geographic distance. Geographic distances between different sampling sites were calculated with the geopy.distance.geodesic function in Python geopy v2.0.0 package^69^ (https://github.com/geopy/geopy). Pearson correlation coefficients were calculated with the cor function in R and plotted using ggpubr^70^. In addition to morphological identification, we conducted species delimitation analysis using the clustering-based method bPTP^71^ based on both the mitochondrial and nuclear trees. We also applied the similarity-based ABGD^72^ and the multispecies coalescent (MSC) model-based BPP v4.3.8^73^ analysis using only the mitochondrial data. We also applied both the A10^74,75^ and A11^76^ analyses implemented in BPP to the 31 *Neodon* specimens using mitochondrial data. Specimens of each morphological species were grouped into the same population, including 1 to 5 specimens of each morphological species. Species delimitation were performed with a user-specified guide tree in the A10 analysis, while species delimitation and species tree inference were jointly calculated in the A11 analysis (detailed in Supplementary Note 1).

### Phylogenetic inference

Both coalescent and concatenation methods were used to infer phylogenetic trees. We inferred the best maximum likelihood trees using RAxML v8.2.12^77^ with the GTR + GAMMA model from 20 independent tree searches and 500 bootstrap replicates for each gene, and then obtained the final species tree using ASTRAL-III^78^ based on the multispecies coalescent model with the bootstrap support of each node being estimated by the multilocus resampling method^79^. SVDquartets (parameters of “eval Quartets = 1e + 6 bootstrap = standard”) implemented in PAUP v4.0a167^80,81^ was also utilised to estimate the species tree with the same dataset to validate the results. Additionally, we concatenated the gene alignments to generate a “supergene” alignment for each species and obtained species trees using IQ-tree v1.6.12^82^, RAxML or MrBayes^83^. The inferred phylogenetic tree comprised two gene sets - the “mitochondrial Gene Set” and the “nuclear Gene Set”. All trees inferred from the nuclear dataset showed the same topology, and we thus re-estimated the branch lengths of the final species tree in units of substitutions per site using ExaML v3.0.21^84^.

### Divergence time estimation

We estimated the divergence times of lineages of *Neodon* based on the second codon sites of nuclear genes using MCMCTree, a Bayesian relaxed clock method implemented in the PAML v4.9 h package^30^. For estimation, an approximate likelihood calculation of the ‘REV’ (GTR, model = 7) model was applied, and multiple fossil calibration points taken from records in the Palaeobiology Database (Accessed 2018 Dec 12)^85^ and the timetree database^86^ were included, as follows: (a) the root age was set as 7.9 Mya, as supported by the occurrence of *Promimomys* in the fossil record;^87^ (b) the splits of *Lasiopodomys* and *Neodon*, *Eothenomys* and *Myodes* were calibrated as <0.53 Mya based on the earliest occurrence of the oldest fossil record of *Myodes* from the Paleobiology Database and another fossil record of *Promimomys*;^87^ (c) the split data of *Eothenomys* was set as 2.7–5.3 Mya based on the fossil calibration point of *Eothenomys* (3.6–2.6 Mya);^88,89^ and (d) the split data of *Microtus* and *Alexandromys* was dated between 0.6 and 3.5 Mya based on previous studies^90,91^. The minimum boundary was supported by the earliest occurrence of *Allophaiomys* in the fossil record in the database. BaseML first estimated a prior substitution rate, and MCMCTree then generated the Gradient and Hessian matrices with following settings: ‘correlated rates clock’ (clock = 3), overall substitution rate (rgene gamma) set of G (1, 12.0), and rate drift parameter (sigma2 gamma) set of G (1, 4.5). Next, we conducted two independent MCMC runs with different random seed numbers and a burn-in of 500,000 iterations to check for convergence. Each run was sampled every 1000 iterations until 500,000 samples had accumulated. We also applied Tracer v1.7.1^92^ to examine the convergence of the MCMC analysis.

### Evolutionary and biogeographic analyses

We scanned for the presence of incomplete lineage sorting (and/or gene flow) spanning the evolution of *Neodon* with the nuclear dataset using DiscoVista^78^. We then calculated the correlation between the content of the non-main topologies of gene trees and the inner-node branch length using a linear model and Pearson’s test in R^93^ and visualised the results using ggplot2^57^. We generated log-lineage through time (LTT) plots for both the time-calibrated phylogeny and 100 simulated trees with the same age and taxonomic richness using Phytools^94^. We used BioGeoBears v1.1.2^95^ for biogeographic reconstruction based on the species tree (detailed in Supplementary Note 1) and assigned the species to one or two of the following biogeographical regions according to their distributions, were the Tsangpo River and the Mekong-Salween rivers divide were used as the borders for E-H and H-D, respectively: P (the QTP); E (Eastern Himalayan Mountains); H (Himalayas); D (Hengduan Mountains); O (area outside of the THR).

### Putative molecular adaptation

Genomes of the low-elevation species *Microtus fortis, Oryctolagus cuniculus, Mus musculus, Rattus norvegicus, Myodes glareolus, Mesocricetus auratus, Neotoma lepida* and *Marmota monax* were downloaded from the Ensembl database (release 101) and used as background taxa in the evolutionary rate analysis. We obtained an orthologous gene set using a BLAST reciprocal best hits (RBH) method^96^, and we then obtained multiple sequence alignments using the same method described above. Next, we retained genes present in all taxa that met the following criteria for analysis using CODEML implemented in PAML v4.9j:^30^ (1) mapping to human gene with at least 50% coverage; (2) lacking frameshift indels in coding sequences; and (3) lacking premature stop codons.

Genes with Bonferroni-adjusted *P* values ≤ 0.05 in the evolutionary rate analysis were treated as candidates that have undergone positive selection. To minimise the influence of alignment errors, positively selected sites were removed when: (1) they appeared as a gap in >2 species; (2) showed >2 forms of nonsynonymous substitutions; and (3) showed probability scores generated by Bayes Empirical Baye analysis (PAML) < 0.9. We further manually checked each PSG to avoid potential false-positives caused by low-quality alignment. The gene functions of the PSGs were annotated using the SwissProt and MGI databases, and KOBAS v3.0^97^ was applied to perform KEGG and GO enrichment annotation. Protein structure was predicted using SWISS-MODEL^98^.

### Statistics and reproducibility

The morphometric variation in non-sex-related measurements of adult specimens was analysed using PCA in SPSS v17.0. We employed Kaiser-Meyer-Olkin and Bartlett’s tests to check the fitness of the PCA, followed by Tukey’s test. Independent-samples two-sided *t* tests or Wilcoxon rank-sum tests were also performed to check the differences between the taxon pairs after PCA. The significant positively selected genes were confirmed using Bonferroni test. Reproducibility was confirmed by performing analyses with independent replicates (for morphological analyses), five hundred bootstrap replicates or different coalescent and concatenation approaches as described in the Methods section.

### Nomenclatural Acts

This published work and the nomenclatural acts it contains have been registered in ZooBank, the proposed online registration system for the International Code of Zoological Nomenclature (ICZN). The ZooBank LSIDs (Life Science Identifiers) can be resolved and the associated information viewed through any standard web browser by appending the LSID to the prefix “http://zoobank.org/”. The LSID for this publication is: urn:lsid:zoobank.org:pub:794808AA-EA46-4E86-B482-9983214688BB.

The LSID for *Neodon namchabarwaensis* Liu SY., Zhou CR., Murphy WR. & Liu SL., sp. nov. is: urn:lsid:zoobank.org:act:8B19E76E-2E5F-452E-A94B-0824DB45CB30

The LSID for *Neodon shergylaensis* Liu SY., Zhou CR., Murphy WR. & Liu SL., sp. nov. is: urn:lsid:zoobank.org:act:811C522A-2B13-48EE-A8B4-3B758E3EB129

The LSID for *Neodon liaoruii* Liu SY., Zhou CR., Meng GL. & Liu SL., sp. nov. is: urn:lsid:zoobank.org:act:D4E07979-F92F-4825-BA6B-BB5B6881F9FD

The LSID for *Neodon bershulaensis* Liu SY., Zhou CR., Liu Y. & Liu SL., sp. nov. is: urn:lsid:zoobank.org:act:A72C6927-1269-4E65-8183-6F48D86F06E9

The LSID for *Neodon bomiensis* Liu SY., Zhou CR., Meng GL. & Liu SL., sp. nov. is: urn:lsid:zoobank.org:act:445E9955-1D43-41E9-AE51-71A3CDFDB28D

The LSID for *Neodon chayuensis* Liu SY., Zhou CR., Liu Y., Tang MK. & Liu SL., sp. nov. is: urn:lsid:zoobank.org:act:0F26DDC2-C279-4DE6-AAF2-B1E4C9917B6F

### Reporting summary

Further information on research design is available in the Nature Research Reporting Summary linked to this article.

## Supplementary information

Supplementary Information - clean version

Description of Additional Supplementary Files

Supplementary Data 1-10

Supplementary Data 11-15

Supplementary Data 16

Supplementary Data 17

Supplementary Data 18-19

Supplementary Data 20

Reporting summary

## Data Availability

Data that support our findings have been deposited in the NCBI database under BioProject PRJNA564473 (ncbi.nlm.nih.gov/bioproject/?term= PRJNA564473) and CNGB Nucleotide Sequence Archive (CNSA) under the accession number CNP0000173 (https://db.cngb.org/search/project/CNP0000173). Source data for figures can be found in Supplementary Data 20.

## Code availability

All analyses were performed using open source software tools, and the detailed parameters for each tool are shown in the relevant methods. Custom scripts are archived at GitHub (https://github.com/linzhi2013/busco_process_scripts).
