# Supplementary material for: Evolution and diversification of Mountain voles (Rodentia: Cricetidae)
Source: Commun Biol. 2022 Dec 26;5:1417. doi: 10.1038/s42003-022-04371-z (PMC9792541; doi:10.1038/s42003-022-04371-z)
Supplement: Supplementary file 1 — Supplementary Information - clean version [file 42003_2022_4371_MOESM1_ESM.pdf]

SUPPLEMENTARY INFORMATION FOR

Evolution and diversification of mountain voles (Rodentia: Cricetidae)

Shaoying Liu[1, *, #] Chengran Zhou[2, 3, *], Guanliang Meng[4], Tao Wan[1], Mingkun Tang[1], Chentao Yang[2], Robert W. Murphy[5, 6], Zhenxin Fan[3], Yang Liu[1], Tao Zeng[3], Yun Zhao[3], Shanlin Liu[7, #]

[1] Sichuan Academy of Forestry, No.18, Xinhui xilu, Chengdu 610081, China

[2] BGI-Shenzhen, Shenzhen 518083, China

[3] Key Laboratory of Bio-Resource and Eco-Environment of Ministry of Education, College of Life Sciences, Sichuan University, Chengdu 610065, China

[4] Zoological Research Museum Alexander Koenig, D-53113 Bonn, Germany

[5] Reptilia Sanctuary and Education Centre, 2501 Rutherford Rd., Concord, ON L4K 2N6

[6] Centre for Biodiversity and Conservation Biology, Royal Ontario Museum, 100 Queen's Park, Toronto, Ontario M5S 2C6, Canada.

[7] Department of Entomology, College of Plant Protection, China Agricultural University, Beijing 100193, China

[*] These authors contributed equally

[#] Corresponding authors: Shaoying Liu, E mail: shaoyliu@163.com, and Shanlin Liu, E mail: shanlin.liu@cau.edu.cn.

THIS PDF FILE INCLUDES

Supplementary Note 1: Supporting Online Materials
Supplementary Note 2: Keys for Species Identification and Expanded Description of New Species
Supplementary Figures
List of Supplementary Appendices
References

SUPPLEMENTARY NOTE 1: SUPPORTING ONLINE MATERIAL

1 Preparation of phallic samples

2 Gene set construction and validation of methods

3 Phylogeny and species delimitation

4 Hypothesis of speciation and evolution for *Neodon*

1 Preparation of phallic samples

We recorded morphological characteristics of male genitalia [1,2], prepared the glans penis using standard methods [1,3], and characterized bacula structures [1,4,5] (Supplementary Data 3). Glans were preserved in 75% ethanol in the field. Dissection was made using a binocular microscope, and morphology of the urethral lappet, dorsal papilla, and outer crater papilla was observed and described. Preparation of bacula followed established methods[1,3]. Morphology of the proximal, distal, and lateral bacula was viewed under a binocular dissecting microscope.

2 Gene set construction and validation of methods

Gene set construction

Read mapping and consensus calling. We obtained orthologous genes for each sample using a reference-based method that mapped short reads onto the reference genome and then obtained the corresponding genomic regions via genome variance (SNP and InDel) identification and consensus-calling functions in bcftools v.1.8 [6,7] with the following criteria: 1) minimum mapping quality of 20; 2) minimum per-Base Alignment Quality (BAQ) of 30; 3) minimum read number for indel candidates of 5; 4) correctly paired reads; 5) optical duplicates and supplementary alignments skipped using default parameter; 6) SNP p-value of 1e-3 in –m mode; and 7) filtering SNPs within 3 bp of an indel and filtering clusters of indels separated by 10 or fewer bp allowing only one to pass. We masked heterozygous sites and sites where the number of high-quality mapped reads (DP4 value) was smaller than 1 to 'Ns'. Parameters were set as "samtools mpileup --max-depth 10000 --min-ireads 5 --redo-BAQ --min-BQ 30 --min-MQ 20 --rf 2 --ff 0x400 --ff 0x800 -f $ref --BCF $bam | bcftools call -m -P 1e-3 | bcftools filter -e 'GT="het"' -g3 -G10 -Oz -o $vcf" (our default filtering parameters).

Gene set construction with the "de novo-derived" method. To test the robustness of the reference-mapping-based ("mapping-derived") method for obtaining single-copy orthologous genes, we assembled six high-coverage samples (Supplementary Data 15) using SOAPdenovo v2.04 r240 with a k-mer size of 31 [8], then performed BUSCO (v3.0.2) to obtain single-copy orthologs ("*de novo*-derived" genes) with database "euarchontoglires odb9". Limited data volume (e.g., depth <10X) could have generated a fragmented assembly that inevitably produced incomplete genes and

missed many of genes. Hence, BUSCO analysis alone could have produced an incorrect gene annotation when genes were missed in the assembly. We calculated K2P distances for gene pairs between the reference genes from *N. shergylaensis* sp. nov. and those obtained from the *de novo* method (Supplementary Fig. 17), and then removed genes with extremely high K2P distances (>20%) from subsequent analyses. Finally, genes without internal stop codons were used to examine the performances of "mapping-derived" method as detailed in the following sections.

Gene set construction using the "mapping-derived" method. In addition to the aforementioned six samples, we sub-sampled two of them (*N. namchabarwaensis* sp. nov. and *Microtus ilaeus*) to test the performance of the "mapping-derived" method for samples with different sequencing depths (depth coverage: 1X, 2X and 5X). We also used the "mapping-derived" gene construction analyses with the following filtering parameters to examine their effects: 1) minimum depth of 5 ("-e DP>=5) ("filDP" in short); 2) minimum DP4 values for reference or alternative locus of 2 ("(DP4[0]+DP4[1])>2 || (DP4[2]+DP4[3])>2") ("filDP4" in short); and 3) minimum quality of 30 and minimum DP4 value of 2 ("-e ((DP4[0]+DP4[1])>2 && ((DP4[2]+DP4[3])<(DP4[0]+DP4[1]))) || (QUAL>30 && ((DP4[2]+DP4[3])>2 && ((DP4[2]+DP4[3])>(DP4[0]+DP4[1]))))") ("filDP4QUAL").

Gene consistency. K2P distances were calculated for each gene pair (*de novo* method vs. mapping method) using the R ape package. We used paired exons of each gene for calculation because of the potential for incongruent exon composition between genes obtained using the *de novo* versus the "mapping-derived" methods. The *de novo* method could have obtained incomplete genes or missed exons due to mis-annotation. The exon pairs were identified using a like-reciprocal best hit (like-RBH) search method. Briefly, for each gene pair, we used MAFFT (v7.313) [9] to perform all-exons-to-all-exons alignments and defined the paired exon as the two exons with reciprocal best match (smallest p-distance) and of a similar length (length difference ≤ 1%, accounting for potentially missed indels using the mapping-derived method).

Validation of the reference-based method

Both species used for testing had 14 gene datasets, including one with default parameters and genome coverage, and six with various filtering cutoffs and genome coverage. As expected, higher genome coverage and a lenient filtering cutoff

produced more genes with a higher level of completeness. The gene datasets of *Microtus ilaeus, Neodon namchabarwaensis* sp. nov., *N. fuscus*, *N. medogensis*, *N. leucurus,* and *Alexandromys limnophilus* were 464,333 bp, 250,984 bp, 545,339 bp, 1,012,979 bp, 566,441 bp and 549,237 bp long, representing 540, 312, 548, 949, 558 and 531 genes, as identified from mapping-derived genes (genome coverage > 10x and default filtering cutoff) and *de novo*-derived genes (Supplementary Data 18). The comparative analysis detected only 24 (0.0052%), 4 (0.0016%), 12 (0.0022%), 14 (0.0014%), 11 (0.0019%), and 4 mismatches (0.0026%), respectively. Lower genome coverage had little effect on sequence accuracy but produced considerably less informative gene sequences. For example, a genome coverage reduction from 10X to 1X for *M. ilaeus* lowered the number of comparable gene pairs from 540 to 13 (exon number from 2,354 to 37) with a minute increase of mismatch rate from 0.0052% to 0.0089% (Supplementary Data 19). Strict filtering cutoffs could have further improved the sequence accuracy but at the expense of gene completeness (details in Supplementary Data 19). Therefore, our results showed that the mapping-derived method generated an effective gene set without introducing any bias in the following phylogenetic analyses.

3 Phylogeny and species delimitation

Morphological analysis

The Euclidean metrics of molar teeth number were calculated between taxa (Supplementary Fig. 2, Supplementary Data 4) and morphological comparisons involved 17 non-gender-related measurements of external, cranial, and dental characteristics of adults (Supplementary Data 5). Fitness testing for the measurements from a PCA analysis obtained a Kaiser-Meyer-Olkin value of 0.941 and a Bartlett's test of < 0.001, demonstrating the robustness of the inference. Thirteen measurements (LM, ZB, SGL, MB, SBL, SH, LIL, ABL, MM, CBL, LMxT, LMbT, and HBL) contributed 60.65% to PC1, and four measurements (TL, IOW, EL, and HFL) contributed 21.48% to PC2. The resulting PCA analysis (Supplementary Fig. 3) and

ANOVAs for the scores of PC1 (F = 45.720, P < 0.001) and PC2 (F = 53.848, P < 0.001) exhibited significant differences among most of the described species of *Neodon*. The contribution of each morphological variable to the principal components in PCA analysis was plotted by R FactoMineR, factoextra, and corrplot [10-12] (Supplementary Fig. 4a). Tukey's post hoc tests also revealed that PC1 or PC2 scores differentiated most taxa. Two-sided *T-tests* (data with normal distributions) or two-sided *Wilcoxon*-tests (data deviated from normal distributions) obtained at least two significantly different measurements in all one-to-one comparisons between taxa not separated in the PCA analysis (Supplementary Fig. 4b).

The smallest interspecific genetic distance of 3.55% for *cox1* (3.70% for *cytb*) occurred between *N. chayuensis* sp. nov. and *N. bomiensis* sp. nov. A clear physical boundary did not separate these species, but their first upper molar differed, and features of their bacula and glans penis differed.

Phylogenetic inference and biogeographic analysis

We downloaded cytochrome b (*cytb*) and cytochrome c oxidase subunit I (*cox1*) sequences, the two most widely used genetic markers for small mammals, from GenBank (accessed in Oct. 2018) for *Neodon* and closely related genera. We obtained data involving 75 *cox1* and 101 *cytb* submissions and used these to infer a phylogenetic tree using the method detailed in the main text. Analyses were conducted for each gene independently owing to differences among included specimens. The gene trees exhibited some conflicts. For example, the most recent common ancestor of *N. irene*, *N. forresti,* and *N. linzhiensis* rooted at the base of the *cox1* tree, but the ancestor of *N. medogensis* and *N. clarkei* did so in the *cytb* tree. More details on the other trees were placed in Supplementary Figs. 7, 10, and 11.

We generated Log-Lineage through time (LTT) plots for both the time-calibrated phylogeny (non-*Neodon* species were pruned) and 100 simulated trees with the same age and taxonomic richness using Phytools [13]. Tree simulation was performed using 'pbtree' implemented in Phytools. For this, the Yule model outperformed the birth-death model in the Akaike information criterion (AIC) test, and a speciation of 0.5520 estimated using 'fit.bd' was utilized for the constant rate model.

We used BIOGEOBEARS v1.1.2 [14] for biogeographic reconstruction based on the species tree and the extended outgroups were pruned from the tree so that only *Neodon*, *Alexandromys*, *Microtus,* and *Lasiopodomys* were analyzed. The maximum range size was set to 2 because no extant species occurred in ≥ 3 biogeographical regions as defined here. Biogeographical reconstructions included dispersal-extinction cladogenesis (DEC) [15], dispersal-vicariance analysis (DIVA) [16], and the BayArea model [17], plus all three models separately under the possibility of founder events (+J) [18,19]. AIC scores were used to compare the fit of different models, and DEC+j was the best-fit model.

Species delimitation

We applied both the A10 [20,21] and A11 [22] analyses implemented in BPP to the 31 specimens of *Neodon*. Specimens of each morphological species were grouped into the same population, resulting in from 1 to 5 specimens for each morphological species. The A10 analysis used a user-specified guide tree, while the A11 analysis jointly performed species delimitation and species tree inference. For the A10 analysis, we used both prior 0 (speciesmodelprior = 0) and prior 1 (speciesmodelprior = 1) and the starting tree (Fig. 3) was constructed via the "nuclear Gene Set" and ASTRAL-III [23] (see the "*Phylogenetic inference*" section). For the A11 analysis, multiple priors were tested (speciesmodelprior = 0, 2, or 3). Population size parameters (qs) were assigned an inverse-gamma prior IG (3, 0.02) with a mean of 0.01 = 0.02/(3 – 1). The root age of the species tree (t0) was assigned an inverse-gamma prior IG(3, 0.005) with a mean of 0.0025, while other divergence-time parameters were specified by uniform Dirichlet distributions [20] (equation 2). The step lengths for the MCMC algorithm were automatically adjusted by the program (finetune=1), starting with the default initial settings. Because the rjMCMC algorithms in BPP for species delimitation were shown to have occasional mixing problems, for each prior setting (the *speciesmodelprior* option) we applied the species delimitation rjMCMC algorithm0 and algorithm1, respectively, and performed two independent runs for each rjMCMC treatment. Each BPP used 20,000 or 100,000 iterations as burn-in, and we took 100,000 MCMC samples every 50 or 100 iterations. Finally, we applied ggplot2 (version 3.3.3) [24] and ggtree (version 2.5.2) [25] packages for convergence diagnosis and visualizations of the MCMC of all BPP runs.

Mitochondrial and nuclear trees were used to estimate species number based on the gene set using three independent methods: ABGD [26], bPTP with Bayesian supported solution (bPTP-BI) [27] and BPP (v4.3.8) [28], of which the ABGD and BPP methods used only the mitochondrial dataset due to their limitation or computational inefficiency. The ABGD method delimited 29 species including outgroup taxa, and supported all the novel morphological species, but also split *N. sikimensis* into two. Meanwhile, bPTP-BI obtained two splits in *N. forresti* and *N. irene*, thus delimiting 31 species. The results from bPTP-BI with the nuclear trees from IQtree also resolved 15 putative species of *Neodon*, but RAxML obtained only 13 species (*N. liaoruii* and *N. shergylaensis* sp. nov. were resolved as a single species). Overall, both A10 and A11 analyses confidently supported the 15 *Neodon* species model, including 6 new species (Supplementary Figs. 7-10). All 12 A11 analysis runs had high posterior possibilities (minimum: 0.87, maximum: 0.99, average: 0.94) for the 15 *Neodon* species model. The A10 analysis results were shown in Supplementary Fig. 8 with high posterior possibilities (median >0.95) for internal branches.

3 Hypothesis of speciation and evolution for the *Neodon*

The time-calibrated genomic analysis (Fig. 3), combined with the geological and climatic events (Fig. 3b), provides important clues for understanding evolutionary patterns in *Neodon*. Thus, we propose the following hypothesis of speciation and evolution for *Neodon*.

Neodon leucurus and *N. fuscus*, the first branch on the tree, occupy the plateau as did the ancestor of *Neodon* (Fig. 4b, bold yellow arrows). The next branch on the southern edge of the QTP around the Himalayas evolved into *N. nyalamensis* and *N. sikimensis*. The third branch represents a dispersal to the southeastern margin of the region and this lineage evolved into *N. medogensis* (Fig. 4b, thin yellow arrows). Formation of the Yarlung Zangbo River created a barrier to the dispersal of *Neodon*, isolating *N. leucurus* and *N. fuscus* in most of the northern part of the plateau with *N. nyalamensis* and *N. medogensis* occupying the narrow area on the south of the Yarlung Zangbo River separated by the Palung Zangbo Ancient River Channel in the eastern and western areas [29] (Supplementary Figs. 14-16). Accordingly, the QTP appears to be the origin and evolution center for *Neodon* and with subsequent dispersal events to the Himalayas, the eastern Himalayas, and Hengduan Mountains.

Following *Neodon*'s dispersal events, climate change and the formation of rivers and mountains led to rapid species radiation. At the early stage of this rapid radiation, the Himalayas, Kangchenjunga Peak, and Namcha Barwa Peak isolated the ancestors of the three major lineages. During the glacial stage (~1.6 MYA), large-scale ice sheets formed on the plateau's surface, and this drove some populations of *Neodon* to relatively warm and humid refugia in the southeastern plateau. This resulted in the origins of *N. linzhiensis, N. namchabarwaensis* sp. nov., *N. liaoruii* sp. nov., and *N. shergylaensis* sp. nov. (Fig. 4b, blue arrows). The Yarlung Zangbo River (and ancient Palon Tsangpo River) separated *N. namchabarwaensis* sp. nov., *N. shergylaensis* sp. nov., and *N. nyalamensis*, while the Duoxiongla Mountain pass (4,200 m a.s.l.) separated the *N. namchabarwaensis* sp. nov. and *N. liaoruii* sp. nov. (Supplementary Fig. 15). This scenario fits well with the concept of sky island effects [30,31]. After forming and connecting with the ancient Palon Tsangpo River, the YLZB River and surrounding mountains (e.g. Galongla Snow Mountain, 4,200 m a.s.l.) at the eastern THR acted as the most important barrier for *Neodon* [29,32]. These barriers, glacial events, and dispersal events then drove more speciation around 0.7 MYA, including the formation of *N. irene*, *N. forresti*, *N. clarkei*, *N. bershulaensis* sp. nov., *N. chayuensis* sp. nov. and *N. bomiensis* sp. nov. (Fig. 4b, orange arrows), which are additional sky island species

The Niyang River did not always function as a barrier to dispersal for all *Neodon* because *N. shergylaensis* sp. nov. and *N. linzhiensis* occur on both sides of this river and the Shergyla Mountain pass (7,728 m a.s.l.). In these cases, glacial events likely broke the original obstructions to gene flow.

SUPPLEMENTARY NOTE 2: KEYS FOR SPECIES IDENTIFICATION AND EXPANDED DESCRIPTION OF NEW SPECIES

For identifying species of *Neodon*, we provide the following key:

1. First lower molar with 5 closed triangles, Sole and palm of feet covering with thick hairs; Or skull very narrow, interorbital width less than 2 mm; Distal bacula long and sturdy: *Lasiopodomys*

First lower molar with 5, 4, or 3 closed triangles; the distal baculum very short: *Neodon* 2

2. First lower molar with 3 closed triangles.. 3

First lower molar with 4 or 5 closed triangles 10

3. TL/HBL approximately 50% .... *N. liaoruii* sp. nov.

TL/HBL less than 45% on average.. 4

4. HBL less than 100mm .... *N. irene*

HBL larger than 100mm.. 5

5. Most specimens with 4 inner and 4 outer angles in the third upper molar .... *N. nyalamensis*

The third upper molar with 4 or 3 inner and 3 outer angles 6

6. The first lower molar with 5 inner and 3 outer angles.. *N. leucurus*

The first lower molar with 4 or 5 outer angles.. 7

7. The second upper molar with 2 inner and 3 outer angles .... *N. forresti*

The second upper molar with 3 inner and 3 outer angles 8

8. The first upper molar with 4 inner and 3 outer angles.. *N. namchabarwaensis* sp. nov.

The first upper molar with 3 inner and 3 outer angles.. 9

9. TL/HBL approximately 44% .... *N. sikimensis*

TL/HBL less than 38% on average.. *N. shergylaensis* sp. nov.

10. The first lower molar with 4 closed triangles 11

The first lower molar with 5 closed triangles 14

11\. The second upper molar with 2 inner and 3 outer angles .... *N. fuscus*

The second upper molar with 3 inner and 3 outer angles 12

12\. The third upper molar usually with 4 inner and 3 outer angles ....*N. medogensis*

The third upper molar with 3 inner and 3 outer angles... 13

13\. The first upper molar of the majority of specimens with 4 inner and 3 outer angles .... *N. chayuensis* sp. nov.

The first upper molar with 3 inner and 3 outer angles..............*N. bomiensis* sp. nov.

14\. TL/HBL larger than 50%.. *N. clarkei*

TL/HBL less than 50% 15

15\. The second upper molar with 2 inner and 3 outer angles .... *N. linzhiensis*

The second upper molar with 3 inner and 3 outer angles.. .... *N. bershulaensis* sp. nov.

Family Cricetidae Rochebrune, 1883

Subfamily Arvicolinae Miller, 1906

Genus *Neodon* Horsfield, 1841

***Neodon namchabarwaensis* Liu SY., Zhou CR., Murphy WR. & Liu SL., sp. nov.**

Namchabarwa Mountain vole

Measurements of Holotype.—The external and cranial measurements (in mm) as follows (abbreviations see Supplementary Data 3): HBL121.0 mm; TL51.0 mm; HFL 21.0 mm; EL 15.0 mm; SGL 27.88 mm; SBL 26.74 mm; CBL 27.70 mm; ZB 15.36 mm; IOW 3.62 mm; MB 12.46 mm; SH 10.60 mm; ABL7.36mm; LMxT 6.12 mm; LMbT 6.44 mm; LM 19.87; M-M 5.48 mm; and OLLI 9.18 mm. Body mass 44g. Photos of the skull, dentition, and mandible are in Supplementary Fig. 1a.

Additional specimens of N. namchabarwaensis sp. nov. —Twenty-two specimens (6 males and 16 females). Nine adults with skulls broken (3♂♂, 6♀♀), field numbers: XZGB0817006♂, XZGB0819001♂, XZGB0820001♂, XZGB0820002♀, XZGB0820003♀, XZGB09N212♀, XZGB09N213♀, XZGB09N214♀, XZGB09N234♀; 13 juveniles (7 intact, 6 with skulls broken; 3♂♂, 10♀♀), field numbers: XZGB0817008♀, XZGB0818007♀, XZGB0818008♀, XZGB0818011♂, XZGB0819002♀, XZGB0819003♀, XZGB0820004♂, XZGB0820005♀, XZGB0820006♀, XZGB0821001♀, XZGB0821002♀, XZGB0821003♂, XZGB0821004♀.

Description.—Appearance same as *N. shergylaensis*. Pelage from head to hip uniform brown-black. Entire back covered with fine, dense, velvety hair. Ventral black-grey, hairs with black base and a very small proportion of yellow-white tip. The transition between dorsal and ventral pelage vague. Ears project above the pelage, covered with short gray-black hairs. Dorsal tail colour black and ventral tail colour lighter; hairs on top of tail slightly longer. Dorsal surface of forefoot and hindfoot yellow-brown. Claws yellow-white. Five palmar and 5 plantar pads. Females with 1 pair of inguinal and pectoral mammae.

Skull sturdy, in dorsal profile straight, and brain case flattened (Supplementary Fig. 1a). Nasal structure broad anteriorly narrowing posteriorly. Parietal elliptic protruding laterally. Interparietal broad, rectangular, middle of anterior part protruding forward. Interorbital and temporal ridges present. Zygomatic arches slender and the middle part slightly broader. Auditory bullae moderately sized. Incisory foramen short and narrow. Posterior palate typical of *Microtus*, with 2 obvious lateral pits. Foramen in palatine very rare. Mandibles medium-sized.

Upper incisors orange. Molars rootless. 1st upper molar with 4 closed triangles after the anterior transverse space, and the last one protruding lingually forwards, 3 outer and 4 inner angles. 2nd upper molar with 3 tooth rings and a posterior-interior small tooth ring after the anterior transverse space, forming 3 inner and 3 outer angles. 3rd upper molar with 3 closed triangles and a "C" tooth loop after the anterior transverse space, this tooth has 4 inner and 3-4 outer angles (Supplementary Fig. 1a6). 1st lower molar with 3 closed triangles in front of the posterior transverse space and a semicircular anterior tooth cap; this tooth has 6 inner and 5 outer angles. 2nd and 3rd lower molars with 3 outer and 3 inner angles (Supplementary Fig. 1a7).

Glans penis (Fig. 2b, Clade 2, Line 3) pole-like and slender with a ventral groove. Outer crater with 2-4 obvious papillae on both sides. Urethral lappet with 3 forks, middle fork shorter. Dorsal papilla with 2 tips, some specimens with 1 tip. Proximal baculum bony with a rhombus-shaped base, and the anterior bulged. Distal baculum also bony and dagger-shaped. Lateral bacular processes bony, bending and relatively longer.

Reproduction.—In early June, most adult males show orchidoptosis, but females are not pregnant. In mid-August, approximately 25% of adult females are pregnant, with 4 embryos, and 50% of males show orchidoptosis. No other data on reproduction are available.

Habitat.—This species inhabits spruce and fir forests at elevations 3160–3700 m a.s.l., with a tree height of approximately 18 m, and 50% coverage. Shrubs 2–3 m and with 20% coverage. Understory humus 5–10 cm thick and grass 10 cm, with 40% coverage.

Nomenclatural statement.—A LSID number was obtained for the new species (*Neodon namchabarwaensis* sp. nov.):

urn:lsid:zoobank.org:act:8B19E76E-2E5F-452E-A94B-0824DB45CB30

***Neodon shergylaensis* Liu SY., Zhou CR., Murphy WR. & Liu SL., sp. nov.**

Shergyla Mountain vole

Measurements of Holotype.—External and cranial measurements (in mm) as follows (abbreviations see Supplementary Data 3): HBL117.0 mm; TL 41.0 mm; HFL 20.0 mm; EL 15.0 mm; SGL 28.22 mm; SBL 26.41 mm; CBL 27.41 mm; ZB 15.79 mm; IOW 4.13 mm; MB 12.56 mm; SH 10.60 mm; ABL7.96 mm; LMxT 6.32 mm; LMbT 6.01 mm; LM 19.55; M-M 5.67 mm; and OLLI 8.66 mm. Body mass 44 g. The photos of the skull, dentition, and mandible are in Supplementary Fig. 1b.

Additional specimens of *N. shergylaensis* sp. Nov.—Twenty-four specimens (13 males and 11 females). Six adults with skulls broken (4♂♂, 2♀♀), field numbers: XZPAR01016♀, XZPAR01023♂, XZBG0802001♂, XZGB09N185♂, XZGB09N186♀, XZGB09N 262♂; 18 juveniles (8 intact, 10 with skulls broken; 9♂♂, 9♀♀), field numbers: LZRAP01010♂, LZRAP01017♂,LZRAP01011♀, LZRAP01015♀, XZGB09N196♀, DJ01001♀, XZGB0802002♀, LZRAP01018♂, LZRAP01021♂, LZRAP01022♀, LZRAP01024♀, LZRAP01025♂, XZGB0801001♀, XZGB0801002♂, XZGB0802001♂, XZGB0816001♂, XZGB09N187♀, XZGB09N198♂.

Description.—Pelage from head to hip uniform brown-black. Entire back covered with fine, dense, velvet hair. Ventral hairs with gray-black base and gray-white tip. Transition between dorsal and ventral pelage vague. Ears project slightly above pelage, covered with dense gray-black hairs. Tail bicolor obvious, dorsal tail brown-black, ventral tail grey-white; hairs on the top of tail slightly longer. Dorsal surface

forefoot and hindfoot yellow-white. Claws yellow-white. Five palmar and 5 plantar pads. Females with 1 pair inguinal and 1 pair pectoral mammae.

Skull sturdy, in dorsal profile straight and brain case flattened. Nasal broad anteriorly narrowing posteriorly. Parietal elliptic and a protrusion on the side. Interparietal broad, anterior part triangle-shaped and posterior margin arc-shaped (Supplementary Fig. 1b). Interorbital and temporal ridges absent. Zygomatic arches slender and middle part slightly broader. Auditory bullae moderately sized. Incisory foramen short and narrow. Posterior palate typical of *Microtus*, with 2 obvious lateral pits. Many small foramens in palatine. Mandibles medium-sized (Supplementary Fig. 1b).

Upper incisors orange. Molars rootless. 1st upper molar with 4 closed triangles after the anterior transverse space, 3 outer and 3 inner angles. 2nd upper molar with 3 closed tooth rings after the anterior transverse space, forming 3 inner and 3 outer angles. 3rd upper molar without closed tooth rings with 4 inner and 3 outer angles (Supplementary Fig. 1b6). 1st lower molar with 3 closed triangles in the front of the posterior transverse space, holotype with 6 inner and 4 outer angles, but 36% of type series with 6 inner and 5 outer angles. 2nd and 3rd lower molars with 3 outer and 3 inner angles (Supplementary Fig. 1b7).

Glans penis (Fig. 2b, clade 2, line 4) pole-like and slender with a ventral groove. Outer crater no obvious papilla. Urethral lappet with 3 forks. Dorsal papilla usually with 1 tip, sometimes with 2 tips. Proximal baculum bony with a rhombus-shaped base. Distal baculum also bony and tongue-shaped. Lateral bacular processes bony, stick-shaped and very short.

Reproduction.—In late May and early of June, most adult males orchidoptosis, but no females pregnant. In mid-September, over 50% of adult females pregnant, with 1–3 embryos, but no male orchidoptosis. No data exist for other months.

Habitat.—This species inhabits fir forest at elevations higher than 3160 m a.s.l., fir height approximately 18 m, 70% coverage. Understory, humus 10–20 cm thick. Moss very abundant, 80% coverage and many fallen dead trees.

Nomenclatural statement.—A LSID number was obtained for the new species (*Neodon shergylaensis* sp. nov.):

urn:lsid:zoobank.org:act:811C522A-2B13-48EE-A8B4-3B758E3EB129

Neodon liaoruii **Liu SY., Zhou CR., Meng GL. & Liu SL., sp. nov.**

Liao's Mountain vole

Measurements of Holotype.—External and cranial measurements (in mm) as follows (abbreviations see Supplementary Data 3): HBL120.0 mm; TL56.0 mm; HFL 21.0 mm; EL 14.0 mm; SGL 29.71 mm; SBL 27.02 mm; CBL 28.67 mm; ZB 15.7 mm; IOW 4.36 mm; MB 12.97 mm; SH 10.44 mm; ABL7.85 mm; LMxT 6.57 mm; LMbT 6.6 mm; LM 20.29; M-M 5.52 mm; and OLLI 9.09 mm. Body mass 42.74 g. Photos of skull, dentition and mandible in Supplementary Fig. 1c.

Additional specimens of N. liaoruii sp. nov.—40 specimens (19 males, and 21 females). 19 adults with skulls broken (9♂♂, 10♀♀), fields numbers: MT11032♂, MT11033♂, MT11034♀, MT11035♀, MT11037♀, MT11054♀, MT11062♂, MT11063♂, MT11065♂, MT11068♀, MT11082♀, MT11083♀, MT11084♀, MT11092♂, MT11094♂, MT11099♀, MT11107♂, MT11145♂, MT11146♀. Twenty-one juveniles (10 ♂♂, 11♀♀; 7 intact, 14 with skulls broken), field

numbers: MT11064♂, MT11069♂, MT11070♂, MT11085♀, MT11093♂, MT11095♂, MT11096♂, MT11097♂, MT11098♀, MT11100♀, MT11101♀, MT11108♂, MT11110♀, MT11111♀, MT11112♀, MT11119♂, MT11121♂, MT11123♀, MM11124♀, MT11125♀, MT11126♀.

Description.—Appearance close to *N. namchabarwaensis*. Pelage from head to hip uniform brown-black. Entire back covered with fine, dense, velvet hair. Ventral hairs yellow-brown with black base. Transition between dorsal and ventral pelage vague. Ears project above pelage, covered with short yellow-brown hairs. Color of back of tail black and ventral of tail lighter; hairs at the top of tail slightly longer. Dorsal surface of forefoot and hindfoot grey-black. Claws yellow-white. Five palmar and 6 plantar pads. Females with 1 pair of inguinal and pectoral mammae.

Skull sturdy, in dorsal profile straight and brain case bulging slightly (Supplementary Fig. 1c1). Nasal broad anteriorly narrowing posteriorly. Parietal trapeziform with a lateral protrusion. Interparietal broad, irregular pentagon (Supplementary Fig. 1c1). Interorbital and temporal ridges presented but weaker. Zygomatic arches slender and middle part slightly broader. Auditory bullae moderately sized. Incisory foramen relatively long and broad. Posterior palate typical of *Microtus*, with 2 obvious lateral pits. Pterygoid with some foramina (Supplementary Fig. 1c2). Mandibles sturdy (Supplementary Fig. 1c5).

Upper incisors orange. Molars rootless. 1st upper molar with 4 closed triangles after the anterior transverse space, 3 outer and 3 inner angles. 2nd upper molar of holotype with 3 closed triangles forming 3 inner and 3 outer angles, but 66% specimens with 2 inner and 3 outer angles. 3rd upper molar of holotype with 4 inner and 3 outer angles, fourth outer angle vestigial; 39% specimens with 3 inner and 3 outer angles. 1st lower molar (Supplementary Fig. 1c6) with 3 closed triangles in front

of the posterior transverse space and a semicircular anterior tooth cap, tooth with 6 inner and 5 outer angles. 2nd and 3rd lower molars with 3 outer and 3 inner angles (Supplementary Fig. 1c7).

Glans penis (Fig. 2b, Clade 2, Line 5) sturdy, pole-like and with a ventral groove. Outer crater with 3-5 inconspicuous papillae on both sides, some specimens no outer crater. Urethral lappet with 3 forks, middle one shorter, sometimes only 2 forks. Dorsal papilla with 2 tips. Proximal baculum bony with a rhombus-shaped base, and concavely forward. Distal baculum bony, tongue-shaped. Lateral bacular processes bony and very short.

Reproduction.—In late October, approximately 10% adult males orchidoptosis, but females not pregnant. No other data on reproduction exist.

Habitat.—Species inhabits mixed coniferous broad leaved forest at elevations about 2700 m a.s.l., tree height approximately 18 m, 40% coverage. Shrubs 4 m and with 20% coverage. Understory, humus 5–10 cm thick and grass 50 cm, 50% coverage.

Nomenclatural statement.—A LSID number was obtained for the new species (*Neodon liaoruii* sp. nov.):

urn:lsid:zoobank.org:act:D4E07979-F92F-4825-BA6B-BB5B6881F9FD

Neodon bershulaensis Liu SY., Zhou CR., Liu Y. & Liu SL., sp. nov.

Bershula Mountain vole

Measurements of Holotype.—External and cranial measurements (in mm) as follows (abbreviations see Data 3): HBL 108.0 mm; TL 55.0 mm; HFL 20.0 mm; EL 14.0 mm; SGL 27.77mm; SBL 26.01mm; CBL 27.19 mm; ZB 15.78 mm; IOW 3.86 mm; MB 12.93 mm; SH 9.90 mm; ABL7.34 mm; LMxT 6.40 mm; LMbT 6.24 mm;

LM 19.90; M-M 5.47 mm; and OLLI 8.18 mm. Body mass35g. Photos of skull, dentition, and mandible in Supplementary Fig. 1d.

Additional specimen of N. bershulaensis sp. nov.—One intact juvenile, field numbers: CHYRD-02-001♂.

Description.—Pelage from head to hip uniform grey-brown. Entire back covered with fine, dense, velvet hair. Ventral hairs grey-white, blushing with brown tinge. Transition between dorsal and ventral pelage vague. Ears project above pelage slightly, covered with short grey-brown hairs. Tail bicolor; dorsum grey-black, ventral grey-white; dorsal hairs slightly longer. Dorsal surface of forefoot and hindfoot grey-black. Claws grey-white, upper surface of nail grey-black. Five palmar and 6 plantar pads. Females with 1 pair of inguinal and pectoral mammae.

Skull relatively sturdy, dorsal profile straight, brain case flattened (Supplementary Fig. 1d). Nasal short, broad anteriorly narrowing posteriorly. Parietal irregular with lateral protrusion. Interparietal broad, irregular rectangle, mid-anterior part protruding forward. Interorbital and temporal ridges absent. Zygomatic arches slender, middle part slightly broader. Auditory bullae moderately sized. Incisory foramen relatively long and broad. Posterior palate typical of *Microtus*, with 2 obvious lateral pits. Many mini-foramen in palate and pterygoid. Mandibles medium (Supplementary Fig. 1d).

Upper incisors orange. Molars rootless. 1st upper molar of holotype with 4 closed triangles after anterior transverse space, 4 outer and 3 inner angles, but inner fourth vestigial; 30% specimens with 3 inner an 3 outer angles. 2nd upper molar with 3 closed triangles after the anterior transverse space, forming 3 inner and 3 outer angles. 3rd upper molar with 4 inner and 3 outer angles (Supplementary Fig. 1d6). 1st lower molar with 5 closed triangles in front of the posterior transverse space and a trefoil

anterior tooth cap, this tooth with 6 inner and 4 outer angles. 2nd and 3rd lower molars with 3 outer and 3 inner angles (Supplementary Fig. 1d7).

Glans penis (Fig. 2b, Clade 3, Line 2) stubby, pole-like and with ventral groove. Outer crater with 1-5 obvious papillae on both sides. Urethral lappet with 2 forks. Dorsal papilla with single tip. Proximal baculum bony with a rhombic-shaped base. Distal baculum bony and triangular. Lateral bacular processes stick-shaped.

Reproduction.—In mid-October and late March, no adult males orchidoptosis and no females pregnant. Other months without reproduction information.

Habitat.—This species inhabits shrubs with sparse firs at elevations of 3450–3750 m a.s.l., tree height approximately 15 m, and 15% coverage. Shrubs 2–3m and with 40% coverage. Understory, humus 10–15cm thick and grass 20–50cm, 20% coverage.

Nomenclatural statement.—A LSID number was obtained for the new species (*Neodon bershulaensis* sp. nov.):

urn:lsid:zoobank.org:act:A72C6927-1269-4E65-8183-6F48D86F06E9

Neodon bomiensis **Liu SY., Zhou CR., Meng GL. & Liu SL., sp. nov.**

Bomi Mountain vole

Measurements of Holotype.—External and cranial measurements (in mm) as follows (abbreviations see Data 3): HBL116.0 mm; TL53.0 mm; HFL 18.0 mm; EL 13.0 mm; SGL 27.56 mm; SBL 25.87 mm; CBL 26.81 mm; ZB 15.89 mm; IOW 4.35 mm; MB 12.53 mm; SH 9.90 mm; ABL7.51 mm; LMxT 6.48 mm; LMbT 6.52 mm; LM 19.96; M-M 5.625 mm; and OLLI 9.10 mm. Body mass 40g. Photos of skull, dentition, and mandible in Supplementary Fig. 4e.

Additional specimens of N. bomiensis sp. nov.—Two specimens with skulls broken, field numbers: MT11305♀, adult; XZ13031♀, juvenile.

Description.—Pelage from head to hip uniform grey-black, brushed with brown-grey tinge. Entire back covered with fine, dense, velvet hair. Ventral hairs grey-white with black base. Transition between dorsal and ventral pelage vague. Ears project above pelage slightly, covered with short grey-black hairs. Tail single color. Dorsal tail black and ventral tail slightly lighter; dorsal tail hairs slightly longer. Dorsal surface of forefoot and hindfoot grey-black. Claws yellow-brown; upper surface of nails grey-black. Five palmar and 5 plantar pads. Females with 1 pair of inguinal and pectoral mammae.

Dorsal profile of skull straight; brain case bulging slightly (Supplementary Fig. 4e). Nasal relatively short, broad anteriorly narrowing posteriorly. Parietal irregular with lateral protrusion. Interparietal broad, irregular elliptic, mid-anterior part protruding forward. Interorbital ridges absent, temporal ridges present but weak. Zygomatic arches slender and middle part slightly broader. Auditory bullae moderately sized. Incisory foramen relatively longer and broader. Posterior palate typical of *Microtus*, with 2 obvious lateral pits. Many mini-foramen in palate and pterygoid. Mandibles medium (Supplementary Fig. 4e).

Upper incisors orange. Molars rootless. 1st upper molar with 4 closed triangles after the anterior transverse space, 3 outer and 3 inner angles. 2nd upper molar of holotype with 3 closed triangles forming 3 inner and 3 outer angles. 3rd upper molar with 4 inner and 3 outer angles (Supplementary Fig. 4e6). 1st lower molar of holotype with 4 closed triangles in front of the posterior transverse space and a trefoil anterior tooth cap; this tooth with 6 inner and 4 outer angles, but 40% specimens with 5 inner and 4 outer angles. 2nd and 3rd lower molars with 3 outer and 3 inner angles (Supplementary Fig. 4e7).

Glans penis (Fig. 2b, Clade 3, Line 4) medium, pole-like, with a ventral groove. Outer crater with 10 obvious papillae on both sides. Urethral lappet with 2 forks. Dorsal papilla with single tip. Proximal baculum bony with a semicircle-shaped base. Distal baculum also bony, short, base bugled largely. Lateral bacular processes cartilaginous and short (Supplementary Fig. 4e).

Reproduction.—In late October and November, no adult males orchidoptosis and no females pregnant. Data not available for other months.

Habitat.—This species inhabits moist mixed coniferous broad leaf forest at elevations about 3150 m a.s.l., tree height approximately 15–-18 m, 60% coverage. Humus 5–10 cm thick. Shrubs 3m and 30% coverage, grass 30 cm height and 15% coverage.

Nomenclatural statement.—A LSID number was obtained for the new species (*Neodon bomiensis* sp. nov.):

urn:lsid:zoobank.org:act:445E9955-1D43-41E9-AE51-71A3CDFDB28D

Neodon chayuensis **Liu SY., Zhou CR., Liu Y., Tang MK. & Liu SL., sp. nov.**

Chayu Mountain vole

Measurements of Holotype.—External and cranial measurements (in mm) as follows (abbreviations see Supplementary Data 3): HBL109.0 mm; TL47.0 mm; HFL 20.0 mm; EL 16.5 mm; SGL 28.01 mm; SBL 26.92 mm; CBL 27.77 mm; ZB 16.46 mm; IOW 3.75 mm; MB 13.02 mm; SH 10.85 mm; ABL7.99 mm; LMxT 6.34 mm; LMbT 6.50 mm; LM 19.95; M-M 5.75 mm; and OLLI 8.97 mm. Body mass 44g. Photos of skull, dentition, and mandible in Supplementary Fig. 1f.

Additional specimens of N. chayuensis sp. nov.—7 specimens (3 males, and 4 females). 5 adults with skulls broken (2♂♂, 3♀♀), field numbers: CY36♀, CY38♂, CY47♂, CY48♀, CY49♀; 2 juveniles, field numbers: CY46♀, CY50♂.

Description.—Pelage from head to hip uniform grey-brown. Entire back covered with fine, dense, velvet hair. Ventral hairs yellow-white with black base. Transition between dorsal and ventral pelage vague. Ears project above pelage slightly, covered with short grey-brown hairs. Tail obviously bicolored. Dorsal color of tail grey-black, ventral yellow-white; hairs at the top of tail slightly longer. Dorsal surface of forefoot and hindfoot grey-black. Claws grey-white, but upper surface of nail black-grey. Five palmar and 5 plantar pads. Females with 1 pair of inguinal and pectoral mammae.

Skull sturdy, in dorsal profile straight and flatten (Supplementary Fig. 1f). Nasal relatively short, broad anteriorly narrowing posteriorly. Parietal irregularly shaped with a lateral protrusion. Interparietal broad, irregularly shaped and the anterior part protruding forward. Interorbital and temporal ridges presented and relatively well-developed. Zygomatic arches relatively sturdy and middle part slightly broader (Supplementary Fig. 1f3). Auditory bullae moderately sized. Incisory foramen relatively long and broad. Posterior palate typical of *Microtus*, with 2 obvious lateral pits. Many foramina in palate pterygoid. Mandibles sturdy (Supplementary Fig. 1f5).

Upper incisors orange, sturdy. Molars rootless. Tooth row sturdy. 1st upper molar with 4 closed triangles after the anterior transverse space, 3 outer and 4 inner angles in holotype; 33% specimens with 3 outer and 3 inner angles. 2nd upper molar with 3 closed triangles forming 3 inner and 3 outer angles. 3rd upper molar without closed triangles, with 4 inner and 3 outer angles (Supplementary Fig. 1f6). 1st lower molar of holotype with 4 closed triangles in front of the posterior transverse space and a trilobal anterior tooth cap, which has 6 inner and 5 outer angles; other 45% specimens

with 6 inner and 4 outer angles. In many specimens, triangles of the 1st lower molar are not closed entirely, more or less confluent each other. 2nd and 3rd lower molars with 3 outer and 3 inner angles (Supplementary Fig. 1f7).

Glans penis (Fig. 2b, Clade 3, Line 5) relatively sturdy and short, pole-like and with a ventral groove. Outer crater with 5 obvious papillae on both sides; also with 2-4 papillae on back crater. Urethral lappet with 3 forks, middle one very short. Dorsal papilla with single tip and sturdy. Proximal baculum bony with rhombic base; middle of bottom concave upward; distal part of proximal baculum expanded as a solder tip. Distal baculum stick-shaped and pointed. Lateral bacular processes cartilaginous and long.

Reproduction.—In early October, approximately 20% adult males orchidoptosis; females not pregnant. No data are available for other months.

Habitat.—This species inhabits marshland at elevations about 3000 m a.s.l. Shrubs 2–3m and with 10% coverage. Grass 40—60 cm, 95% coverage. Humus 10–20 cm thick.

Nomenclatural statement.—A LSID number was obtained for the new species (*Neodon chayuensis* sp. nov.):

urn:lsid:zoobank.org:act:0F26DDC2-C279-4DE6-AAF2-B1E4C9917B6F

SUPPLEMENTARY FIGURES

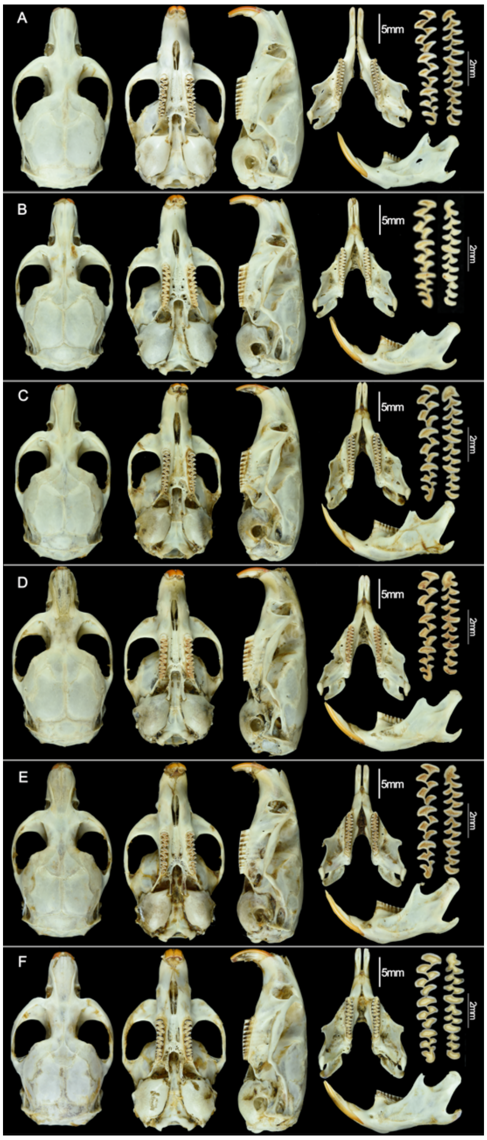

Supplementary Fig. 1 Skull comparisons for the unidentified taxa. 1. Ventral view, 2. Dorsal view, 3. Lateral view, 4. Ventral lower jaw, 5. Lateral lower jaw, 6. Upper toothrow and 7. Lower toothrow of six unidentified taxa of *Neodon*. **a** unidentified taxon 1 (from Nanyi township, Milin County), **b** unidentified taxon 2 (from Shergyla Mountain, Linzhi county), **c** unidentified taxon 3 (from Motuo County, south of Namchabarwa Mountain), **d** unidentified taxon 4 (from Ridong village, Bershula Mountain, Chayu County), **e** unidentified taxon 5 (from Bomi County), **f** unidentified taxon 6 (from Chibagou National Nature Reserve, Chayu County).

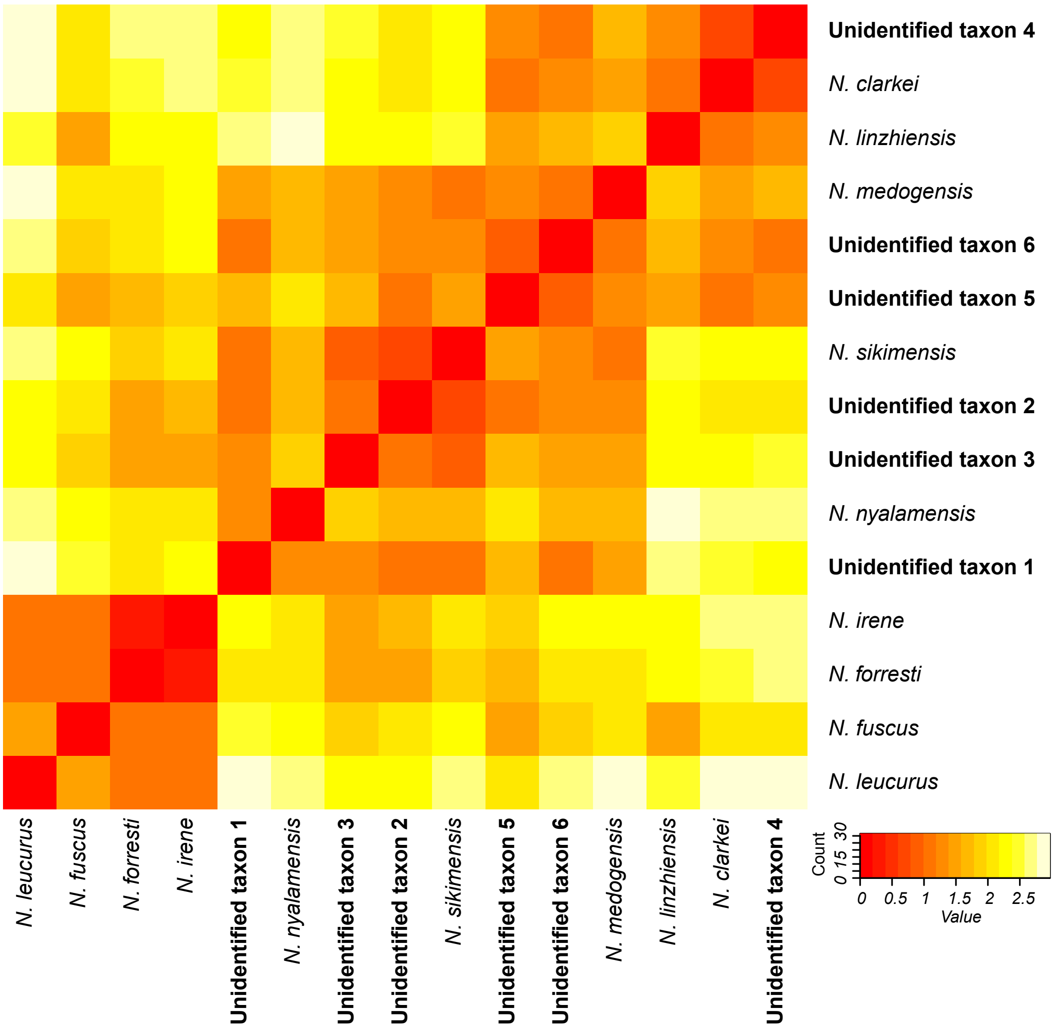

Supplementary Fig. 2 Euclidean distances for quantities from molar teeth. Higher values indicate more differences between pairs.

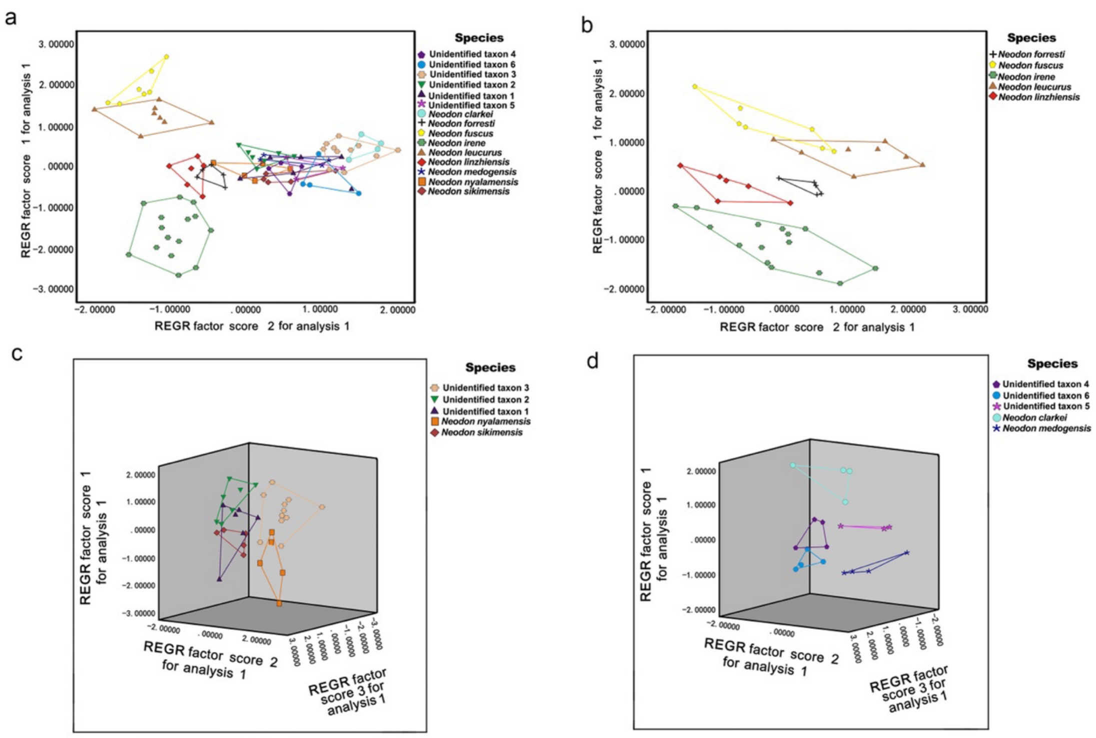

Supplementary Fig. 3 PCA results for the morphological measurements. **a** PCA results for the 17 non-gender-related measurements of all *Neodon* samples. Taxa with different quantities of the closed triangles in the first lower molar were in different panels: **b** with 3 closed triangles in the first lower molar, **c** 4, and **d** 5.

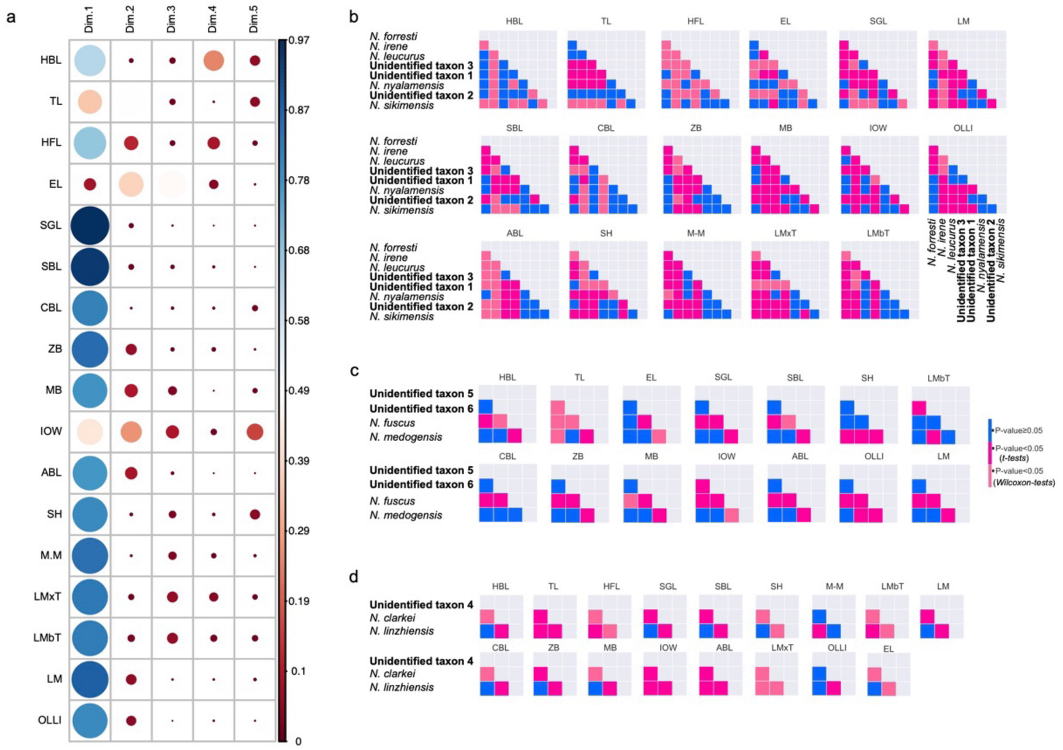

Supplementary Fig. 4 Comparison of morphological measurements. **a** Contribution of each morphological variable to the principal components in PCA analysis. The size and the color of each circle represent the quality of representation for each variable. **b** Two-sided t test results for taxa with three closed triangles in the first lower molar (NM1 value of 3). **c** Two-sided Wilcoxon rank-sum test results for species with a NM1 value of 3. **d** T test results for species with a NM1 value of 4. **e** Wilcoxon-test results for species with a NM1 value of 4. **f** T tests results for species with a NM1 value of 5. **g** Wilcoxon-test results for species with a NM1 value of 5. P-values between species pairs for b-e were divided into two groups: P-value < 0.05 (purple) and P-value≥0.05) (blue), while missing data were represented by grey color. Measurements were on a logarithmic scale and detailed measurements can be found in Supplementary Data 3. Only measurements with significant differential results were shown.

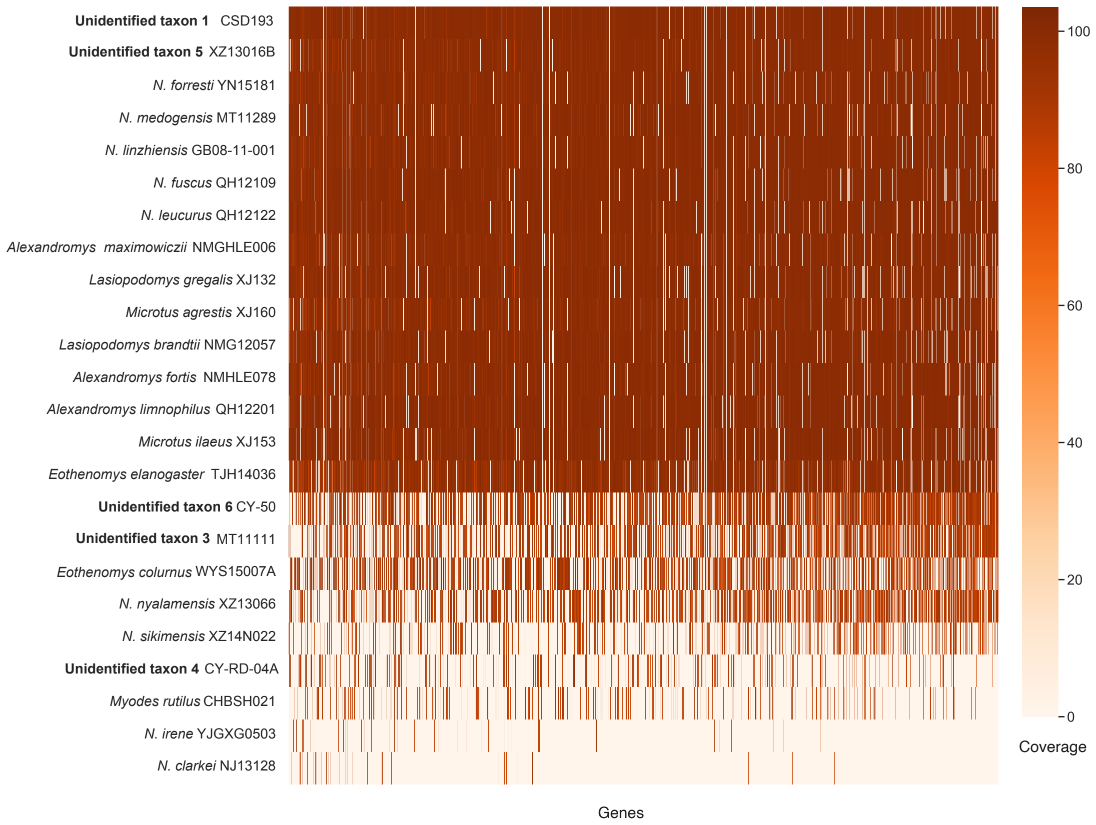

Supplementary Fig. 5 Heatmap of nuclear gene coverage. A total of 4,624 coding genes were obtained in this study. All taxa but two with > 500 genes were used for subsequent analyses (114 and 106 genes for *Neodon irene* and *Neodon clarkei*, respectively).

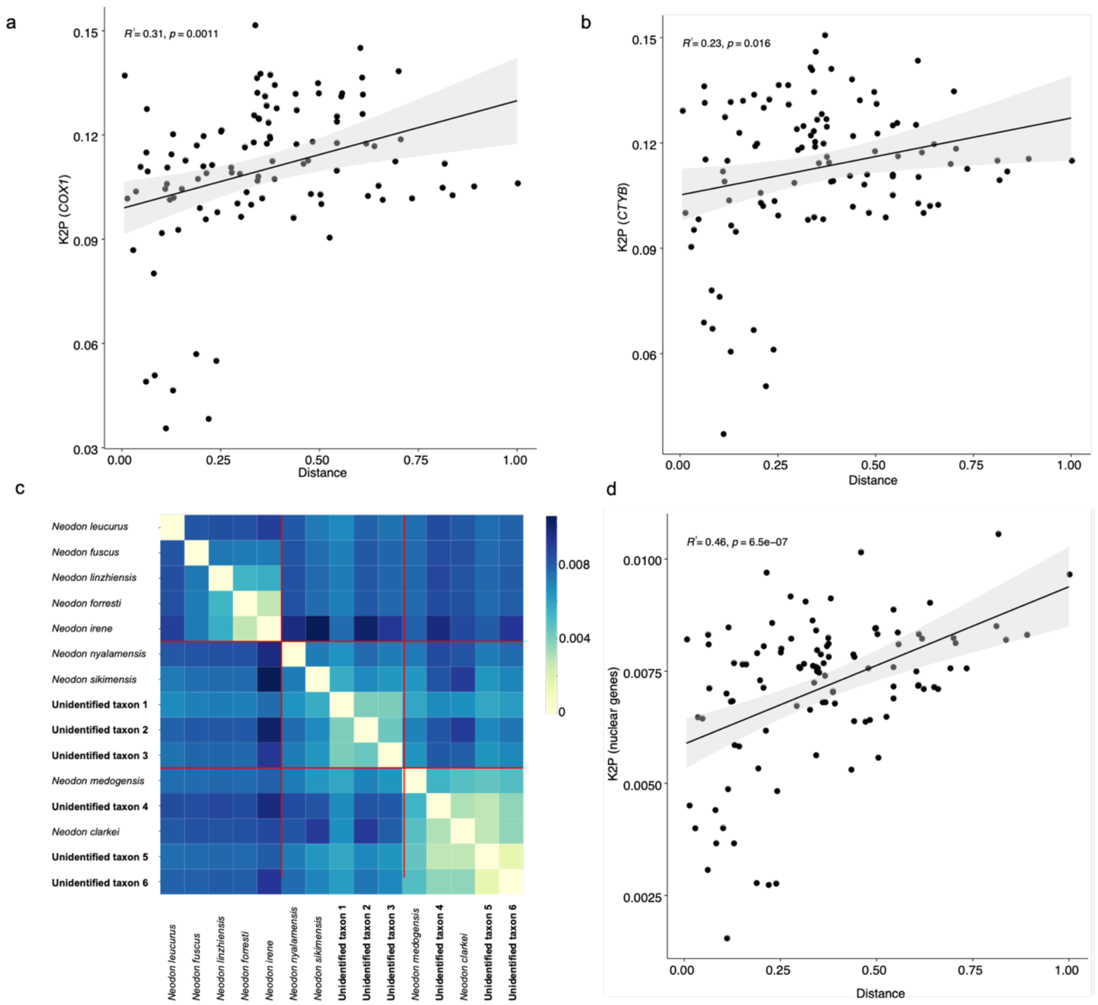

Supplementary Fig. 6 K2P distances for genes between lineages of *Neodon*. **a** Correlation between K2P distances of *cox1* and geographic distances. **b** Correlation between K2P distances of *cytb* and geographic distances. **c** Average genetic distances for all nuclear genes between each pair species shown in this heatmap of species arranged into Clade 1, Clade 2 and Clade 3 by red lines (clade information in Fig. 3). **d** Correlation between average K2P distances of coding regions for nuclear genes and geographic distances.

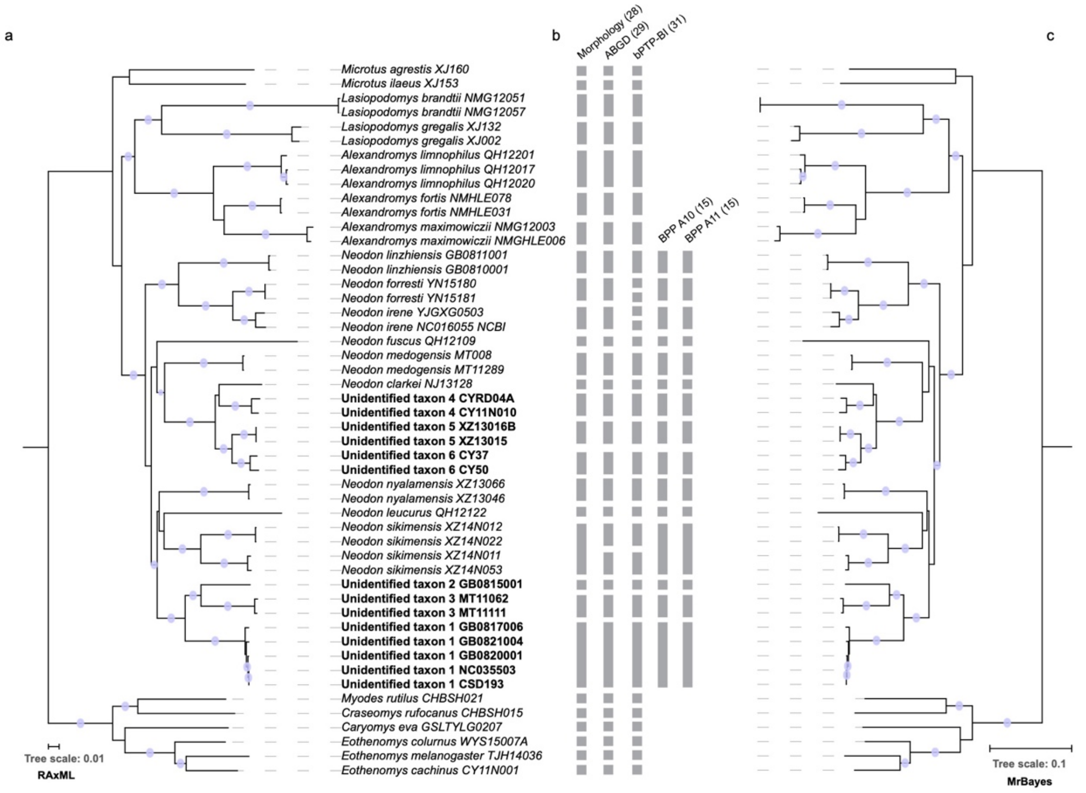

Supplementary Fig. 7 Mitochondrial trees and species delimitation. **a** Mitochondrial phylogenetic tree from RAxML with all mitochondrial coding genes. **b** Species delimitation results, and **c** MrBayes tree with all mitochondrial coding genes. The transparent purple bars represent bootstrap values ≥95 in a and credible intervals ≥95% in **c**.

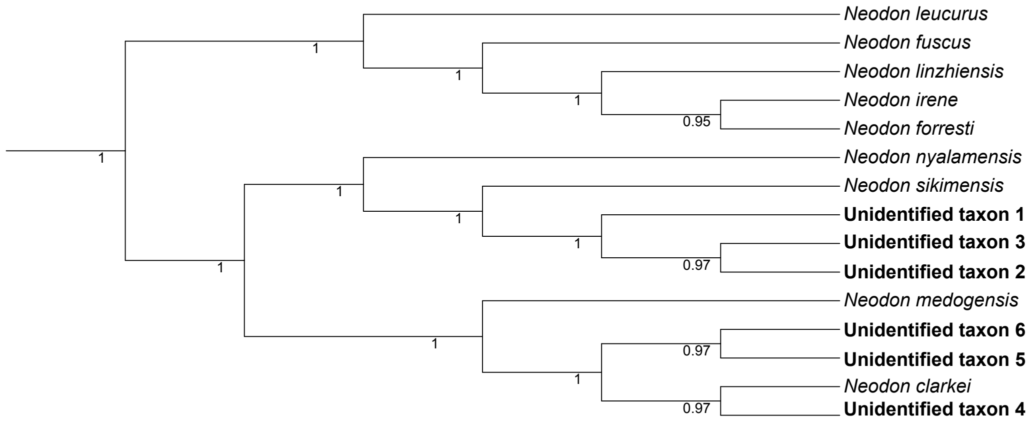

Supplementary Fig. 8 Bayesian posterior possibilities from A10 analysis. Median values of multiple runs shown. Analysis performed with guide tree Fig. 3 and 13 PCGs of these specimens. Different combinations of priors (speciesmodelprior = 0 or 1) and rjMCMC algorithm (0 or 1) were tested, for each combination, and at least two runs were performed. New taxa are indicated by bold type.

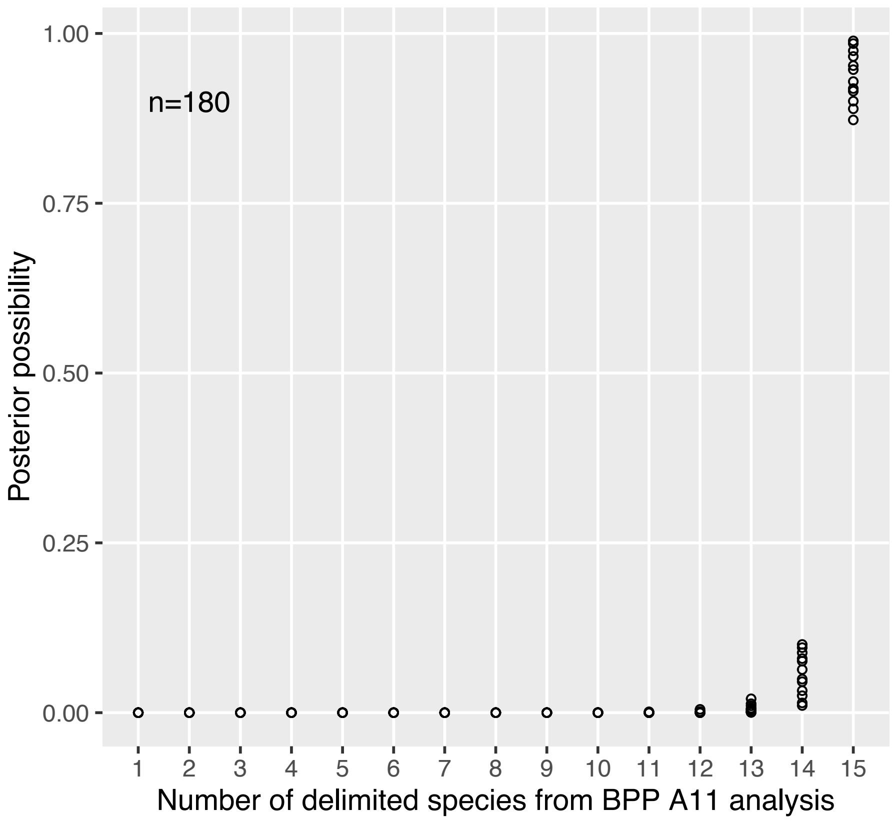

Supplementary Fig. 9 Posterior possibility distribution of BPP A11 Analysis. Different combinations of priors (speciesmodelprior = 0/2/3) and rjMCMC algorithm (0 or 1) were tested, for each combination, and at least two runs were performed.

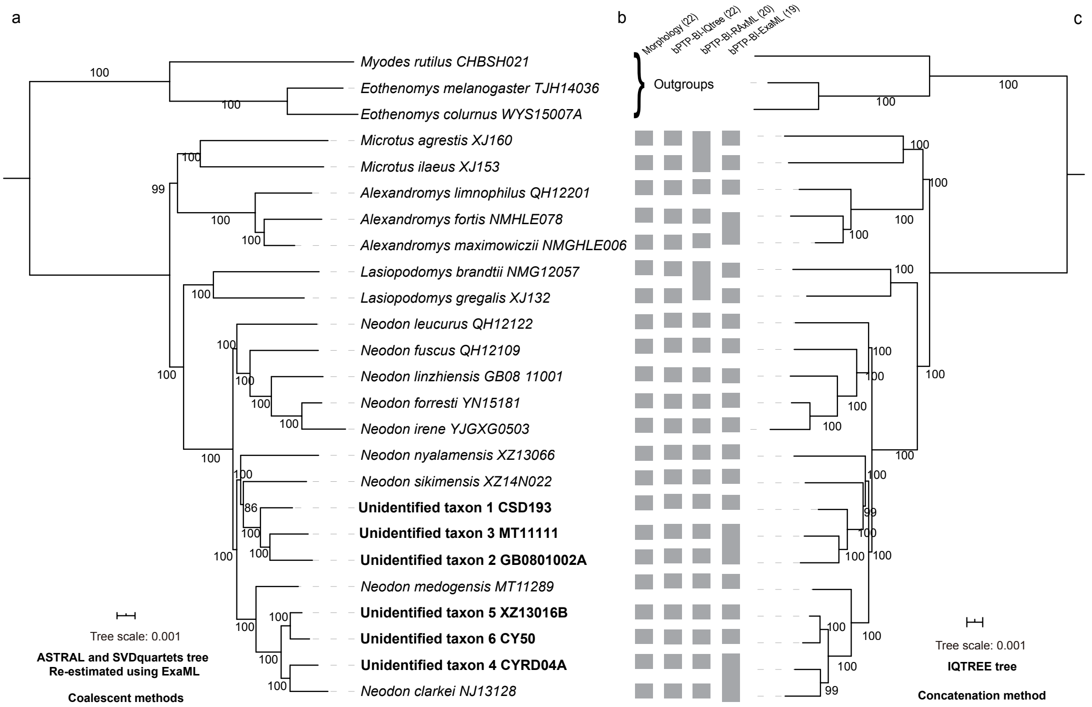

Supplementary Fig. 10 Nuclear DNA trees and species delimitation. **a** Phylogenomic tree obtained using the ASTRAL-III method (with a normalized quartet score of 73.69% and branch support ≥ 86) and SVDquartets with all 4,624 genes dataset. Branch lengths were re-estimated in units of substitutions per site using ExaML with concatenated nuclear genes. **b** Species delimitation results. **c** Phylogenomic tree inferred from IQTree with concatenated nuclear genes (branch supports ≥ 99). The topology was identical using both coalescent-based methods (ASTRAL and SVDquartets) and concatenation methods (RAxML and IQTree).

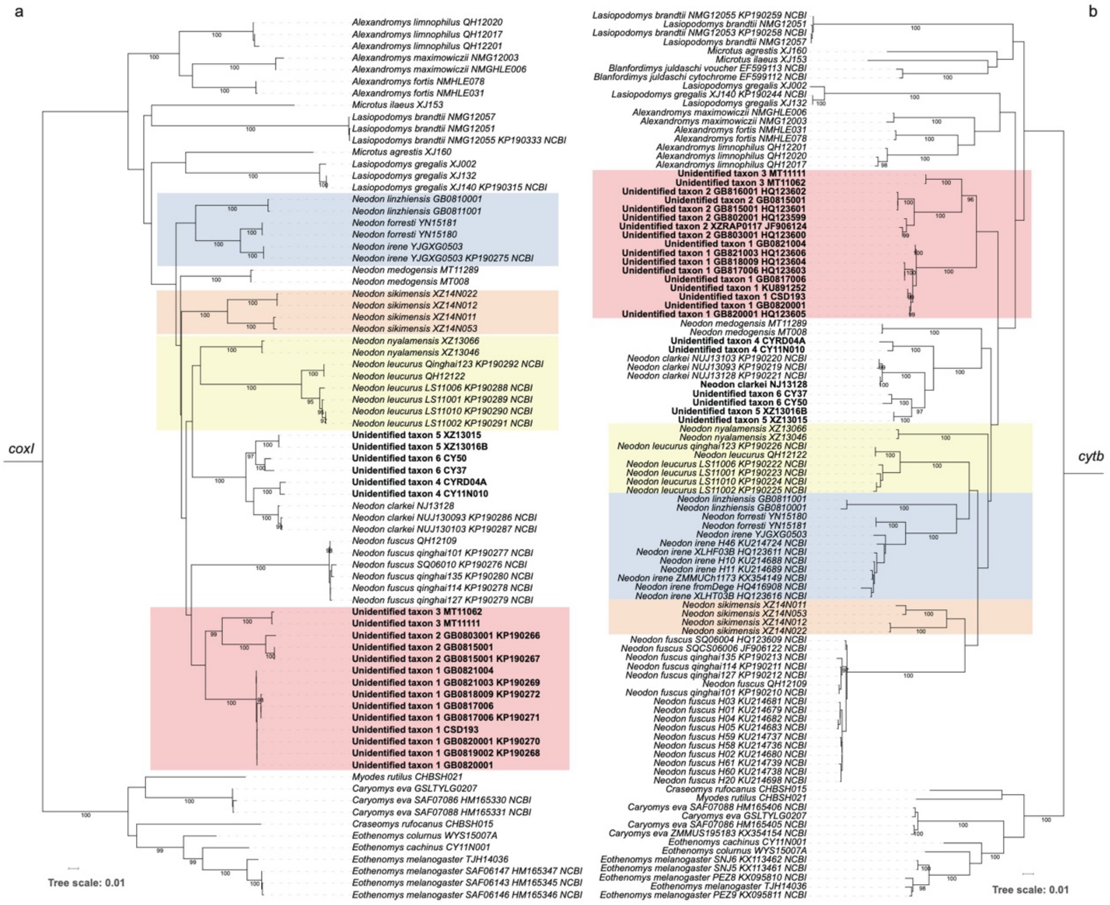

Supplementary Fig. 11 Phylogenetic trees inferred by *cox1* and *cytb* inferred using RAxML. The colored rectangles represent the conflicts between trees. Bootstrap values ≥ 95 were shown near the branches.

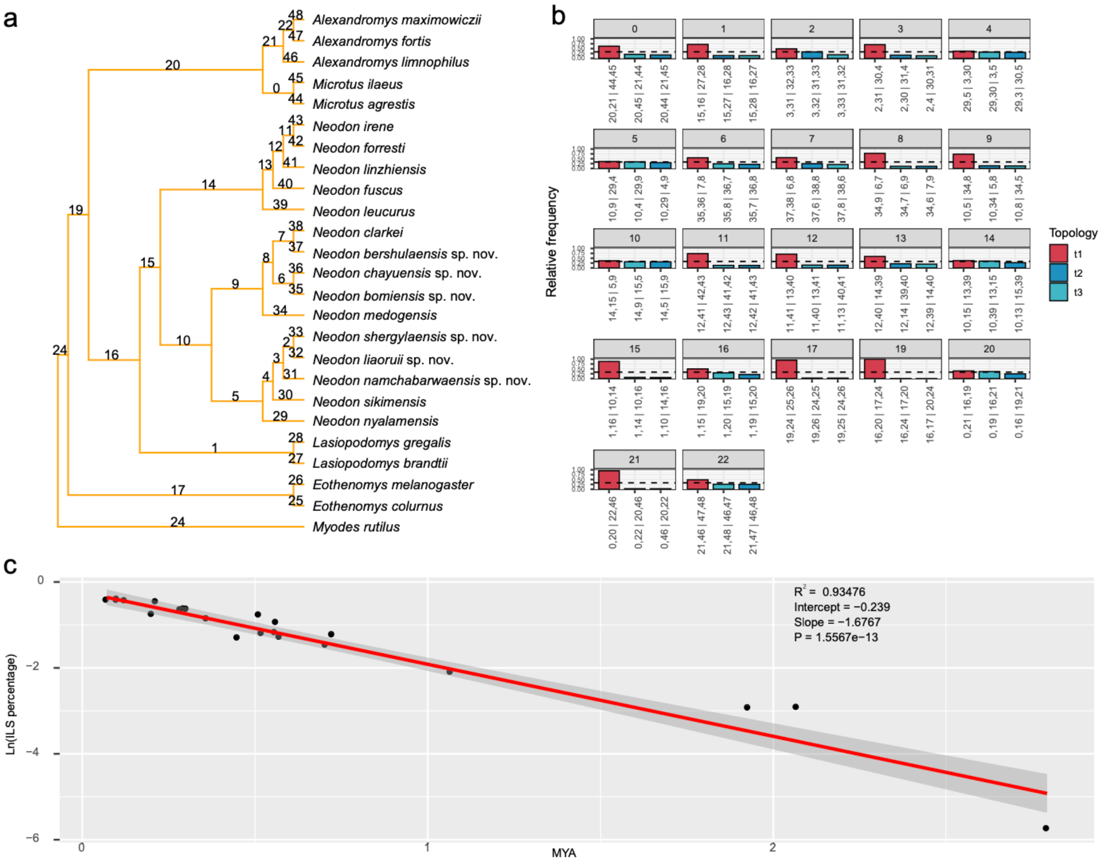

Supplementary Fig. 12 Frequency analysis for the three gene tree topologies. **a** Species tree with the number of each branch. **b** Relative frequency. The title of each subfigure corresponds to an internal branch on the tree. The frequencies of the three topologies around each internal branch were calculated using DiscoVista with gene trees inferred using RaxML. The main topologies are shown in red, and the two alternative topologies are shown in blue. The dotted lines indicate the 1/3 threshold. On the x-axis, the exact definition of each quartet topology is provided using the neighboring branch labels separated by "|". **c** Relationship between ILS occurrence frequency (Ln value) and inner-node branch length of the time tree.

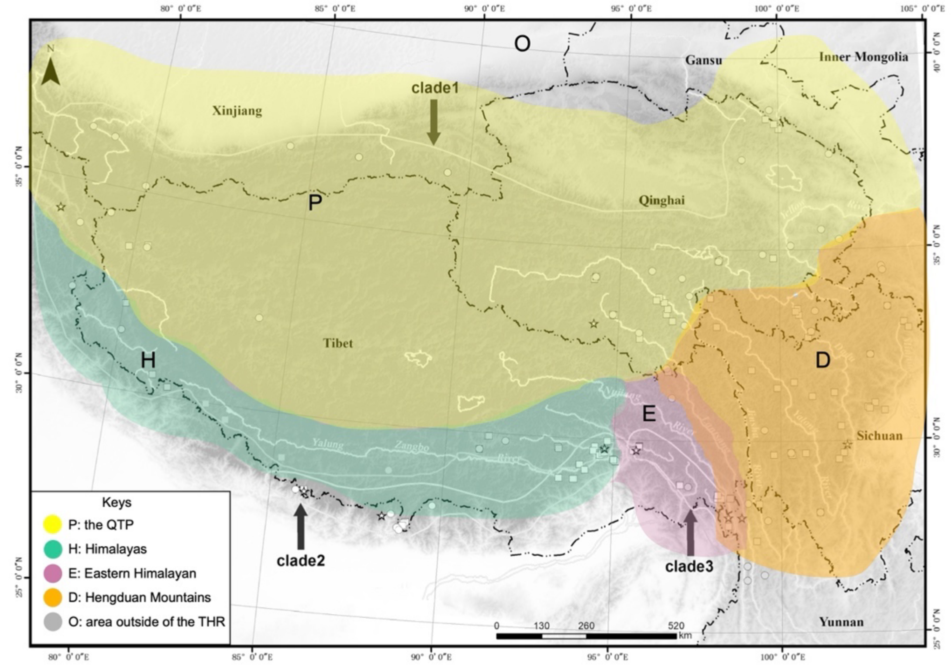

Supplementary Fig. 13 Map of areas used in BioGeoBears analysis

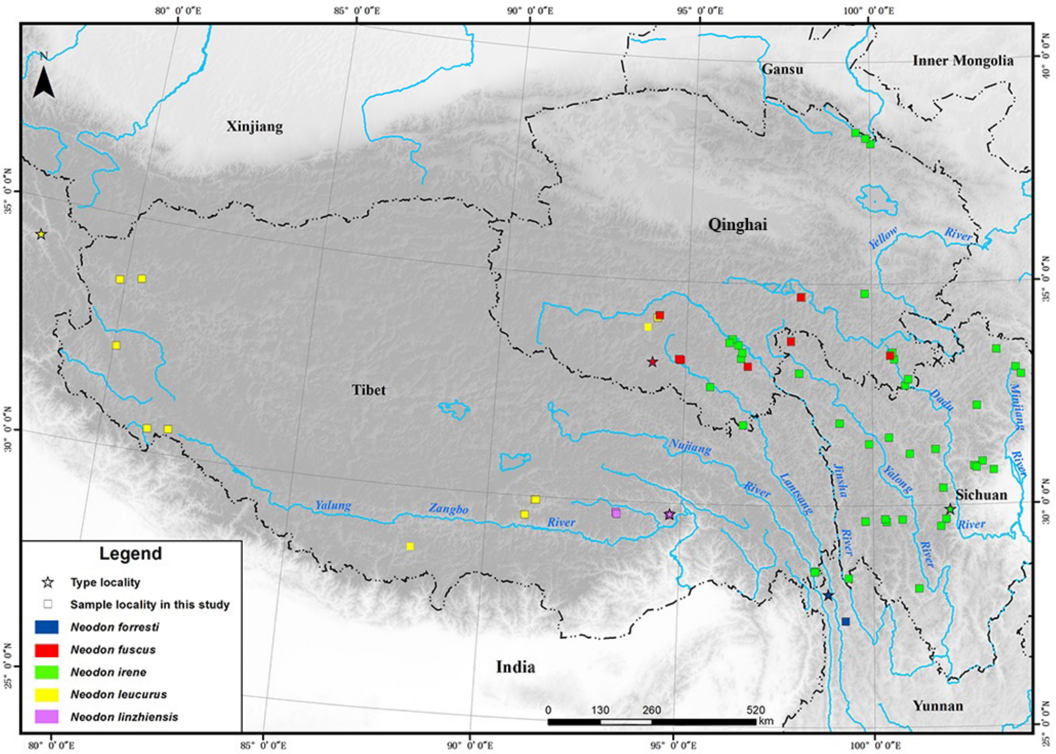

Supplementary Fig. 14 Distribution patterns of five taxa from *Neodon* Clade 1.

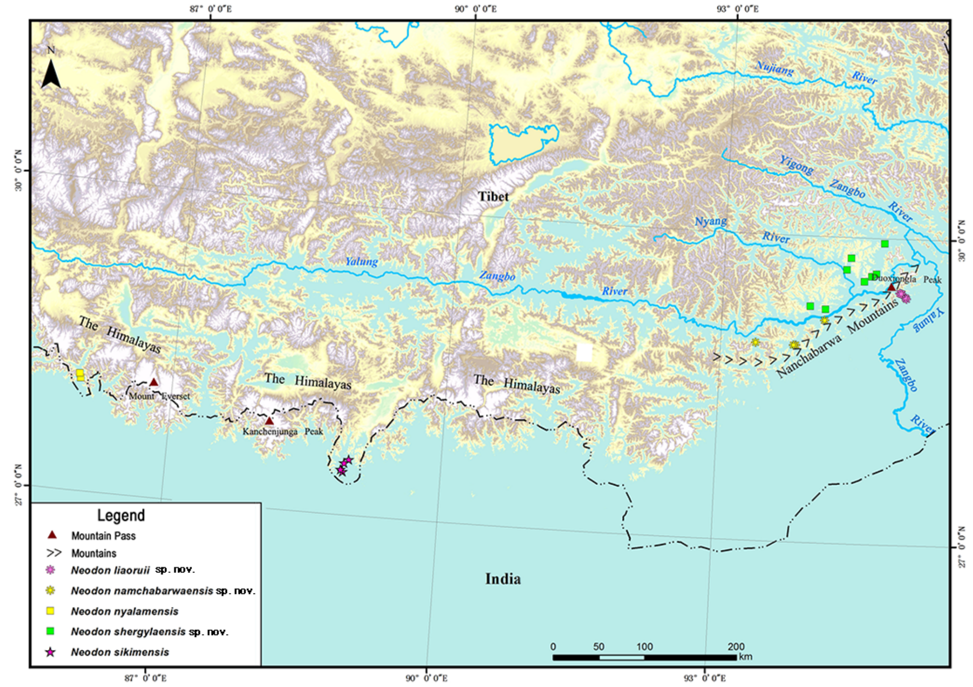

Supplementary Fig. 15 Distribution patterns of five taxa from *Neodon* Clade 2.

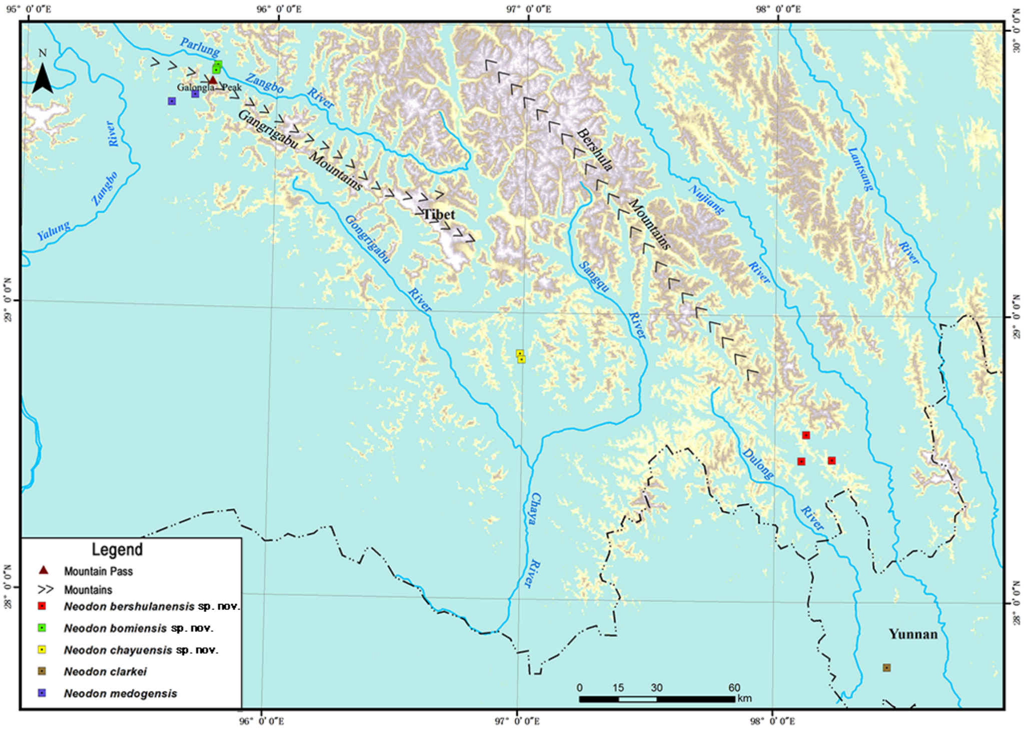

Supplementary Fig. 16 Distribution patterns of five taxa from *Neodon* Clade 3.

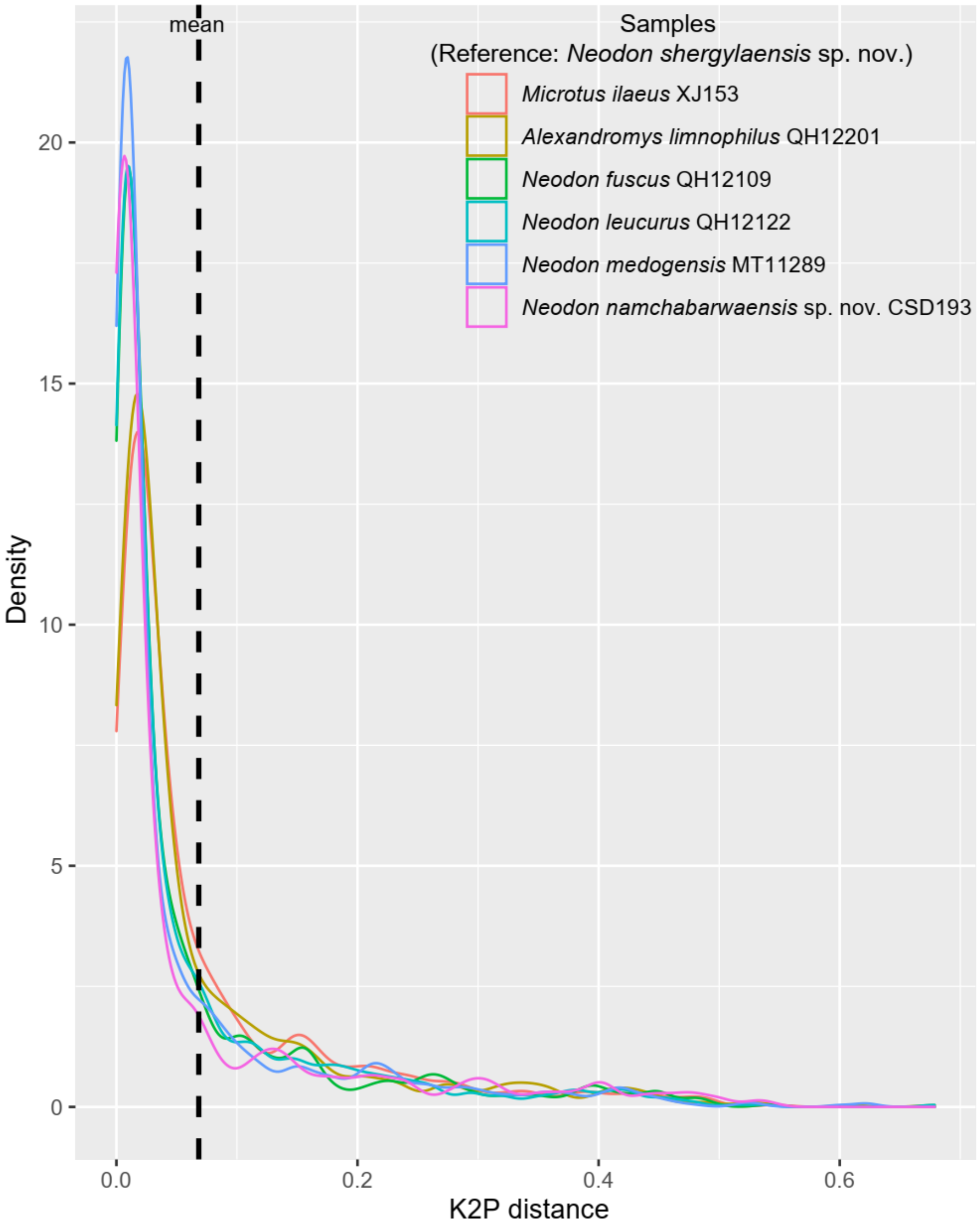

Supplementary Fig. 17 K2P distance density between reference genes and annotated genes from *de novo* method.

LIST OF SUPPLEMENTARY APPENDICES

Supplementary Notes 1-2

Supplementary Figures Supplementary Figures 1-17

Supplementary Data 1-20:

Supplementary Data 1 The number of specimens used in this study

Supplementary Data 2 Information for sequenced samples

Supplementary Data 3 Measurements of morphological characteristics

Supplementary Data 4 Initial morphology comparison of 15 taxa of *Neodon*. Tooth and skull characteristics of 155 intact adults (15 putative species). Abbreviations refer to Supplementary Data 3. Numbers for the tooth characteristics show trait states, for example, *Neodon bershulaensis* sp. nov. has 5 closed triangles in the first lower molar tooth (NM1: 5). Percentages before the numbers show the proportion of individuals with that trait state, for example, "70%: 4; 30%: 3" for the UM1I of *N. bershulaensis* sp. nov. means that 70% individuals have 4 inner angles in the first upper molar tooth, while 30% of the individuals have 3 inner angles in the first upper molar tooth. The last column shows the number of individuals being analyzed for each species.

Supplementary Data 5 Measurements of 17 non-gender-related characteristics for PCA analyses. The tooth and skull characteristics of 95 specimens are shown. Specimens are phylogenetically divided into 3 clades, see Supplementary Fig. 14-16 for details. Abbreviations refer to Supplementary Data 3.

Supplementary Data 6 Shapiro test and Levene test results of morphological characteristics. The null hypothesis that the data were drawn from a normal distribution is tested for each trait of each species. The null hypothesis that all input samples (species 1 vs. species 2) are from populations with equal variances is also tested for each trait of each species. Abbreviations refer to Supplementary Data 3.

Supplementary Data 7 T test and Wilcoxon rank-sum test results of statistical measurements of morphological characteristics. Abbreviations refer to Supplementary Data 3.

Supplementary Data 8 Intra-species distances of 13 mitochondrial protein-coding genes.

Supplementary Data 9 Congenic inter-species distances of 13 mitochondrial protein-coding genes.

Supplementary Data 10 Inter-genera distances of 13 mitochondrial protein-coding genes.

Supplementary Data 11 Fit for DEC and DEC+j models of ancestral range estimates. The best-fit model is the Dispersal-extinction cladogenesis with a long-distance J parameter (DEC+J).

Supplementary Data 12 Positively selected genes from the PAML branch-site model.

Supplementary Data 13 KEGG enrichment results.

Supplementary Data 14 GO enrichment results.

Supplementary Data 15 MGI phenotype annotation.

Supplementary Data 16 Maker control files.

Supplementary Data 17 Phylogenetic results.

Supplementary Data 18 Statistics for genomes and genes used in method comparison. We *de novo* assembled genomes of 6 high-coverage sequencing samples (Library IDs with prefix "CL1000XXX") using SOAPdenovo v2.04 r240 with a k-mer size of 31, then performed BUSCO (v3.0.2) to obtain their single-copy orthologs ("*de novo*-derived" genes) with database "euarchontoglires odb9". Then we calculated the K2P genetic distances of these genes to the reference genes (i.e., *Neodon shergylaensis* sp. nov., RDWHANIccdDAADAA-A2, was from 10X sequencing and assembled with SuperNova v2.1.1). The gene pairs with extremely high K2P distances (>20%) were removed from subsequent comparative analyses.

Supplementary Data 19 Comparison of exon pairs from two methods. We obtained single-copy orthologs of 6 high-coverage sequencing samples using both *de novo* assembly and reference-mapping-based ("mapping-derived") methods. The K2P genetic distance of each gene pair was then calculated using the R ape package. For the reference-mapping-based method, we tested the effect of different sequencing depth (data subsampling: 1X, 2X, 5X) and different VCF file filtering parameters.

Supplementary Data 20 Source data for figures.

REFERENCES

1. Hooper, E. T. *The male phallus in mice of the genus Peromyscus*. Vol. 105 (Museum of Zoology, University of Michigan, 1958).
2. Hooper, E. T. & Hart, B. S. *A synopsis of recent North American microtine rodents*. Vol. 120 (Museum of Zoology, University of Michigan, 1962).
3. Lidicker Jr, W. A phylogeny of New Guinea rodent genera based on phallic morphology. *Journal of Mammalogy* **49**, 609-643 (1968).
4. Yang, A. & Fang, L. Phallic morphology of 13 species of the family Muridae from China, with comments on its taxonomic significance. *Acta Theriologica Sinica* **4**, 275-285 (1988).
5. Yang, A., Liu, S. & Fang, L. Phallic morphology of eight species in Gerbillinae and Microtinae from China. *Acta Theriologica Sinica* **1**, 31-38 (1992).
6. Li, H. *et al.* The Sequence Alignment/Map format and SAMtools. *Bioinformatics* **25**, 2078-2079 (2009).
7. Danecek, P. & McCarthy, S. A. BCFtools/csq: haplotype-aware variant consequences. *Bioinformatics* **33**, 2037-2039 (2017).
8. Luo, R. *et al.* SOAPdenovo2: an empirically improved memory-efficient short-read de novo assembler. *GigaScience* **1**, 2047-2217X-2041-2018 (2012).
9. Katoh, K. & Standley, D. M. MAFFT multiple sequence alignment software version 7: improvements in performance and usability. *Molecular Biology and Evolution* **30**, 772-780 (2013).
10. Kassambara, A. & Mundt, F. Package 'factoextra'. *Extract and visualize the results of multivariate data analyses* **76** (2017).
11. Lê, S., Josse, J. & Husson, F. FactoMineR: an R package for multivariate analysis. *Journal of Statistical Software* **25**, 1-18 (2008).
12. Wei, T. *et al.* Package 'corrplot'. *Statistician* **56**, e24 (2017).
13. Revell, L. J. phytools: an R package for phylogenetic comparative biology (and other things). *Methods in Ecology and Evolution* **3**, 217-223 (2012).
14. Matzke, N. J. BioGeoBEARS: BioGeography with Bayesian (and likelihood) evolutionary analysis in R Scripts. *R package, version 0.2* **1**, 2013 (2013).
15. Ree, R. H. & Smith, S. A. Maximum likelihood inference of geographic range evolution by dispersal, local extinction, and cladogenesis. *Systematic Biology* **57**, 4-14 (2008).
16. Ronquist, F. Dispersal-vicariance analysis: a new approach to the quantification of historical biogeography. *Systematic Biology* **46**, 195-203 (1997).
17. Landis, M. J., Matzke, N. J., Moore, B. R. & Huelsenbeck, J. P. Bayesian analysis of biogeography when the number of areas is large. *Systematic Biology* **62**, 789-804 (2013).

18 Matzke, N. J. Model selection in historical biogeography reveals that founder-event speciation is a crucial process in island clades. *Systematic Biology* **63**, 951-970 (2014).
19 Matzke, N. J. Probabilistic historical biogeography: new models for founder-event speciation, imperfect detection, and fossils allow improved accuracy and model-testing. *Frontiers of Biogeography* **5** (2013).
20 Yang, Z. & Rannala, B. Bayesian species delimitation using multilocus sequence data. *Proceedings of the National Academy of Sciences* **107**, 9264-9269 (2010).
21 Rannala, B. & Yang, Z. Improved reversible jump algorithms for Bayesian species delimitation. *Genetics* **194**, 245-253 (2013).
22 Yang, Z. & Rannala, B. Unguided species delimitation using DNA sequence data from multiple loci. *Molecular Biology and Evolution* **31**, 3125-3135 (2014).
23 Zhang, C., Rabiee, M., Sayyari, E. & Mirarab, S. ASTRAL-III: polynomial time species tree reconstruction from partially resolved gene trees. *BMC Bioinformatics* **19**, 153 (2018).
24 Wickham, H. *ggplot2: elegant graphics for data analysis*. (Springer, 2016).
25 Yu, G., Smith, D. K., Zhu, H., Guan, Y. & Lam, T. T. Y. ggtree: an R package for visualization and annotation of phylogenetic trees with their covariates and other associated data. *Methods in Ecology and Evolution* **8**, 28-36 (2017).
26 Puillandre, N., Lambert, A., Brouillet, S. & Achaz, G. ABGD, automatic barcode gap discovery for primary species delimitation. *Molecular Ecology* **21**, 1864-1877 (2012).
27 Zhang, J., Kapli, P., Pavlidis, P. & Stamatakis, A. A general species delimitation method with applications to phylogenetic placements. *Bioinformatics* **29**, 2869-2876 (2013).
28 Flouri, T., Jiao, X., Rannala, B. & Yang, Z. Species tree inference with BPP using genomic sequences and the multispecies coalescent. *Molecular Biology and Evolution* **35**, 2585-2593 (2018).
29 Chen, J., Ji, J., Gong, J. & Qing, J. Formation of the Yarlung Zangbo Grand Canyon, Tibet, China. *Geological Bulletin of China* **27**, 491-499 (2008).
30 McCormack, J. E., Huang, H., Knowles, L. L., Gillespie, R. & Clague, D. Sky islands. *Encyclopedia of Islands* **4**, 841-843 (2009).
31 Mosbrugger, V., Favre, A., Muellner-Riehl, A. N., Päckert, M. & Mulch, A. *"Cenozoic evolution of geo-biodiversity in the Tibeto-Himalayan region," in Mountains, Climate, and Biodiversity*. (Wiley-Blackwell, 2018).
32 Wang, E.-q., Chen, L.-z. & Chen, Z.-l. Tectonic and climatic element-controlled evolution of the Yalungzangbu River in southern Tibet. *Quaternary Sciences* **22**, 365-373 (2002).
