# Supplementary material for: Evolution and diversification of Mountain voles (Rodentia: Cricetidae)
Source: Commun Biol. 2022 Dec 26;5:1417. doi: 10.1038/s42003-022-04371-z (PMC9792541; doi:10.1038/s42003-022-04371-z)
Supplement: Supplementary file 2 — Description of Additional Supplementary Files [file 42003_2022_4371_MOESM2_ESM.pdf]

Description of Additional Supplementary Files

File name: Supplementary Data 1
Description: The number of specimens used in this study.

File name: Supplementary Data 2
Description: Information for sequenced samples.

File name: Supplementary Data 3
Description: Measurements of morphological characteristics.

File name: Supplementary Data 4
Description: Initial morphology comparison of 15 taxa of Neodon. Tooth and skull characteristics of 155 intact adults (15 putative species). Abbreviations refer to Supplementary Data 3. Numbers for the tooth characteristics show trait states, for example, Neodon bershulaensis sp. nov. has 5 closed triangles in the first lower molar tooth (NM1: 5). Percentages before the numbers show the proportion of individuals with that trait state, for example, "70%: 4; 30%: 3" for the UM1I of N. bershulaensis sp. nov. means that 70% individuals have 4 inner angles in the first upper molar tooth, while 30% of the individuals have 3 inner angles in the first upper molar tooth. The last column shows the number of individuals being analyzed for each species.

File name: Supplementary Data 5
Description: Measurements of 17 non-gender-related characteristics for PCA analyses. The tooth and skull characteristics of 95 specimens are shown. Specimens are phylogenetically divided into 3 clades, see Supplementary Fig. 14-16 for details. Abbreviations refer to Supplementary Data 3.

File name: Supplementary Data 6
Description: Shapiro test and Levene test results of morphological characteristics. The null hypothesis that the data were drawn from a normal distribution is tested for each trait of each species. The null hypothesis that all input samples (species 1 vs. species 2) are from populations with equal variances is also tested for each trait of each species. Abbreviations refer to Supplementary Data 3.

File name: Supplementary Data 7
Description: T test and Wilcoxon rank-sum test results of statistical measurements of morphological characteristics. Abbreviations refer to Supplementary Data 3.

File name: Supplementary Data 8
Description: Intra-species distances of 13 mitochondrial protein-coding genes.

File name: Supplementary Data 9
Description: Congenic inter-species distances of 13 mitochondrial protein-coding genes.

File name: Supplementary Data 10
Description: Inter-genera distances of 13 mitochondrial protein-coding genes.

File name: Supplementary Data 11
Description: Fit for DEC and DEC+j models of ancestral range estimates. The best-fit model is the Dispersal-extinction cladogenesis with a long distance J parameter (DEC+J).

File name: Supplementary Data 12
Description: Positively selected genes from the PAML branch-site model.

File name: Supplementary Data 13
Description: KEGG enrichment results.

File name: Supplementary Data 14
Description: GO enrichment results.

File name: Supplementary Data 15
Description: MGI phenotype annotation.

File name: Supplementary Data 16
Description: Maker control files.

File name: Supplementary Data 17
Description: Phylogenetic results.

File name: Supplementary Data 18
Description: Statistics for genomes and genes used in method comparison. We de novo assembled genomes of 6 high-coverage sequencing samples (Library IDs with prefix "CL1000XXX") using SOAPdenovo v2.04 r240 with a k-mer size of 31, then performed BUSCO (v3.0.2) to obtain their single-copy orthologs ("de novo-derived" genes) with database "euarchontoglires odb9". Then we calculated the K2P genetic distances of these genes to the reference genes (i.e., Neodon shergylaensis sp. nov., RDWHANIccdDAADAA-A2, was from 10X sequencing and assembled with SuperNova v2.1.1). The gene pairs with extremely high K2P distances (>20%) were removed from subsequent comparative analyses.

File name: Supplementary Data 19
Description: Comparison of exon pairs from two methods. We obtained single-copy orthologs of 6 high-coverage sequencing samples using both de novo assembly and reference-mapping-based ("mapping-derived") methods. The K2P genetic distance of each gene pair was then calculated using the R ape package. For the reference-mapping-based method, we tested the effect of different sequencing depth (data subsampling: 1X, 2X, 5X) and different VCF file filtering parameters.

File name: Supplementary Data 20
Description: Source data for figures.
