# Supplementary material for: Evolution and diversification of Mountain voles (Rodentia: Cricetidae)
Source: Commun Biol. 2022 Dec 26;5:1417. doi: 10.1038/s42003-022-04371-z (PMC9792541; doi:10.1038/s42003-022-04371-z)
Supplement: Supplementary file 9 — Reporting summary [file 42003_2022_4371_MOESM9_ESM.pdf]

nature portfolio

Corresponding author(s): Shaoying Liu, Shanlin Liu

Last updated by author(s): Nov 28, 2022

Reporting Summary

Nature Portfolio wishes to improve the reproducibility of the work that we publish. This form provides structure for consistency and transparency in reporting. For further information on Nature Portfolio policies, see our Editorial Policies and the Editorial Policy Checklist.

Statistics

For all statistical analyses, confirm that the following items are present in the figure legend, table legend, main text, or Methods section.

n/a	Confirmed	
☐	☒	The exact sample size (*n*) for each experimental group/condition, given as a discrete number and unit of measurement
☐	☒	A statement on whether measurements were taken from distinct samples or whether the same sample was measured repeatedly
☐	☒	The statistical test(s) used AND whether they are one- or two-sided<br>*Only common tests should be described solely by name; describe more complex techniques in the Methods section.*
☒	☐	A description of all covariates tested
☐	☒	A description of any assumptions or corrections, such as tests of normality and adjustment for multiple comparisons
☐	☒	A full description of the statistical parameters including central tendency (e.g. means) or other basic estimates (e.g. regression coefficient) AND variation (e.g. standard deviation) or associated estimates of uncertainty (e.g. confidence intervals)
☐	☒	For null hypothesis testing, the test statistic (e.g. *F*, *t*, *r*) with confidence intervals, effect sizes, degrees of freedom and *P* value noted<br>*Give P values as exact values whenever suitable.*
☐	☒	For Bayesian analysis, information on the choice of priors and Markov chain Monte Carlo settings
☒	☐	For hierarchical and complex designs, identification of the appropriate level for tests and full reporting of outcomes
☐	☒	Estimates of effect sizes (e.g. Cohen's *d*, Pearson's *r*), indicating how they were calculated

Our web collection on statistics for biologists contains articles on many of the points above.

Software and code

Policy information about availability of computer code

Data collection	No software was used for data collection.
Data analysis	SPSS V17.0, SOAPnuke v 1.5.6, Supernova v2.1.1, SOAPdenovo v2.04r240, BWA vO.7.17, BCFtools v1.8, MAKER2 V2.31.10, MitoZ, GATK v4.2, BLAST v2.6.0+, PSMC v0.6.4-r49, BUSCO v3.0.1, MAFFT v7.3.13, PAL2NAL, TrimAl v1.4.rev22, ASTRAL-III, ExaML v3.0.21, RAxML v8.2.12, IQtree v1.6.12, SVDquartets, PUAP v4.0a167, Tracer v1.7.1, PAML v4.9 (MCMCTREE, BASEML, CODEML), DiscoVista, Sentieon v201911, R v3.6.3, BioGeoBEARS, ggtree, ape, ggplot2, corrplot, ggpubr, PHYTOOLS, PYTHON3, geopy v2.0.0, github.com/geopy/geopy, ABGD, bPTP, BPP V4.3.8, KOBAS v3.0, SWISS-MODEL https://swissmodel.expasy.org/, and custom scripts are archived at GitHub (https://github.com/linzhi2013/busco_process_scripts)

For manuscripts utilizing custom algorithms or software that are central to the research but not yet described in published literature, software must be made available to editors and reviewers. We strongly encourage code deposition in a community repository (e.g. GitHub). See the Nature Portfolio guidelines for submitting code & software for further information.

Data

Policy information about availability of data

All manuscripts must include a data availability statement. This statement should provide the following information, where applicable:

- Accession codes, unique identifiers, or web links for publicly available datasets
- A description of any restrictions on data availability
- For clinical datasets or third party data, please ensure that the statement adheres to our policy

Data that support our findings have been deposited in the NCBI database under BioProject PRJNA564473 (ncbi.nlm.nih.gov/bioproject/?term= PRJNA564473) and CNGB Nucleotide Sequence Archive (CNSA) under the accession number CNP0000173 (https://db.cngb.org/search/project/CNP0000173).

Field-specific reporting

Please select the one below that is the best fit for your research. If you are not sure, read the appropriate sections before making your selection.

☐ Life sciences ☐ Behavioural & social sciences ☒ Ecological, evolutionary & environmental sciences

For a reference copy of the document with all sections, see nature.com/documents/nr-reporting-summary-flat.pdf

Ecological, evolutionary & environmental sciences study design

All studies must disclose on these points even when the disclosure is negative.

Study description	We reported on a collection of specimens of mountain voles taken over the past 20 years from the Tibetan-Himalayan region and revealed their diversification and evolutionary history using both morphological and molecular data: 1) We provided a high-quality Neodon reference genome and generated whole genome sequencing data for each representative morphologically distinct species. 2) Identified six new species, which demonstrates the underestimation of diversity in the Tibetan-Himalayan region. 3) Our analyses reveal that climate change events acted as key factors drivers of diversification and evolution. 4) The genetic basis for the adaptations also illuminate how Tibetan voles have adapted to the plateau environment under different pressures and occupied these niches.
Research sample	In total, 235 Neodon samples were used for morphological analysis and genomes of 48 Glires samples were sequenced in this study.
Sampling strategy	All samples were obtained following Guidelines of the American Society of Mammalogists and the laws and regulations of China for the implementation of the protection of terrestrial wild animals. Collecting protocols and research project were approved by the Ethics Committee of the Sichuan Academy of Forestry and the Institutional of Review Board on Bioethics and Biosafety of BGI (NO. FT17005).
Data collection	The data used in our study includes genome data, which was extracted directly from tissue samples and sequenced.
Timing and spatial scale	Samples used in this study were collected during August 2001 to November 2021, and mainly from in or around the Himalayas, the Hengduan Mountains and the Qinghai-Tibetan Plateau.
Data exclusions	NA
Reproducibility	All attempts to repeat the experiment on the data obtained were successful.
Randomization	NA - Our data consisted of genomes, which were not randomized.
Blinding	Blinding was not relevant to our study as we were only using morphological and genome data.

Did the study involve field work? ☒ Yes ☐ No

Field work, collection and transport

Field conditions	Complex natural environments of high-altitude plateau and river valley, including montane grasslands, alpine desert, tundra, alpine shrub, alpine conifer and mixed forests, etc.
Location	Samples were collected mainly from in or around the Himalayas, the Hengduan Mountains and the Qinghai-Tibetan Plateau (Detailed in Supplementary Fig. 14-16 and Supplementary Data 1-2).
Access & import/export	All samples were obtained following Guidelines of the American Society of Mammalogists and the laws and regulations of China for the implementation of the protection of terrestrial wild animals. Collecting protocols and research project were approved by the Ethics Committee of the Sichuan Academy of Forestry and the Institutional of Review Board on Bioethics and Biosafety of BGI (NO. FT17005).

Disturbance	NA - The samples used in this study were collected as part of a monitoring programme on Tibetan voles, and were integrated in this study several years after they had been collected.

Reporting for specific materials, systems and methods

We require information from authors about some types of materials, experimental systems and methods used in many studies. Here, indicate whether each material, system or method listed is relevant to your study. If you are not sure if a list item applies to your research, read the appropriate section before selecting a response.

Materials & experimental systems

n/a	Involved in the study	
☒	☐	Antibodies
☒	☐	Eukaryotic cell lines
☒	☐	Palaeontology and archaeology
☐	☒	Animals and other organisms
☒	☐	Human research participants
☒	☐	Clinical data
☒	☐	Dual use research of concern

Methods

n/a	Involved in the study	
☒	☐	ChIP-seq
☒	☐	Flow cytometry
☒	☐	MRI-based neuroimaging

Animals and other organisms

Policy information about studies involving animals; ARRIVE guidelines recommended for reporting animal research

Laboratory animals	NA
Wild animals	In total, 235 Neodon samples were used for morphologic analysis and genomes of 48 Glires samples were sequenced in this study. The samples used in this study were collected as part of a monitoring programme on mountain voles, and were integrated in this study several years after they had been collected.
Field-collected samples	NA
Ethics oversight	All samples were obtained following Guidelines of the American Society of Mammalogists and the laws and regulations of China for the implementation of the protection of terrestrial wild animals. Collecting protocols and research project were approved by the Ethics Committee of the Sichuan Academy of Forestry and the Institutional of Review Board on Bioethics and Biosafety of BGI (NO. FT17005).

Note that full information on the approval of the study protocol must also be provided in the manuscript.
